# Antigen Extraction and B Cell Activation Enable Identification of Rare Membrane Antigen Specific Human B Cells

**DOI:** 10.3389/fimmu.2019.00829

**Published:** 2019-04-16

**Authors:** Maria Zimmermann, Natalie Rose, John M. Lindner, Hyein Kim, Ana Rita Gonçalves, Ilaria Callegari, Mohammedyaseen Syedbasha, Lukas Kaufmann, Adrian Egli, Raija L. P. Lindberg, Ludwig Kappos, Elisabetta Traggiai, Nicholas S. R. Sanderson, Tobias Derfuss

**Affiliations:** ^1^Department of Biomedicine, University Hospital Basel, University of Basel, Basel, Switzerland; ^2^Novartis Institute for BioMedical Research, Basel, Switzerland; ^3^BioMed X Innovation Center, Heidelberg, Germany; ^4^Laboratory of Virology, Geneva University Hospitals, Geneva, Switzerland; ^5^Neuroscience Consortium, Monza Policlinico and Pavia Mondino, University of Pavia, Pavia, Italy; ^6^Division of Clinical Microbiology, University Hospital Basel, University of Basel, Basel, Switzerland; ^7^Departments of Medicine, Neurologic Clinic and Policlinic, Clinical Research and Biomedical Engineering, University Hospital and University of Basel, Basel, Switzerland; ^8^Department of Medicine, Neurologic Clinic and Policlinic, University Hospital and University of Basel, Basel, Switzerland

**Keywords:** autoimmunity, membrane protein antigens, trogocytosis, human monoclonal antibodies, myasthenia gravis

## Abstract

Determining antigen specificity is vital for understanding B cell biology and for producing human monoclonal antibodies. We describe here a powerful method for identifying B cells that recognize membrane antigens expressed on cells. The technique depends on two characteristics of the interaction between a B cell and an antigen-expressing cell: antigen-receptor-mediated extraction of antigen from the membrane of the target cell, and B cell activation. We developed the method using influenza hemagglutinin as a model viral membrane antigen, and tested it using acetylcholine receptor (AChR) as a model membrane autoantigen. The technique involves co-culturing B cells with adherent, bioorthogonally labeled cells expressing GFP-tagged antigen, and sorting GFP-capturing, newly activated B cells. Hemagglutinin-specific B cells isolated this way from vaccinated human donors expressed elevated CD20, CD27, CD71, and CD11c, and reduced CD21, and their secreted antibodies blocked hemagglutination and neutralized viral infection. Antibodies cloned from AChR-capturing B cells derived from patients with myasthenia gravis bound specifically to the receptor on cell membrane. The approach is sensitive enough to detect antigen-specific B cells at steady state, and can be adapted for any membrane antigen.

## Introduction

A cardinal characteristic of the humoral immune response is that only a minuscule fraction of the total B cell pool recognizes a given antigen. Understanding these cells is therefore hindered by the practical difficulty of identifying them. The immunoglobulin ELIspot (1) enables quantification of B cells of a given specificity, but for live cell assays, immunoglobulin gene cloning, and single cell technologies such as RNA sequencing, isolation of intact cells is key.

Flow cytometric methods are an obvious solution, enabling immediate *ex vivo* phenotyping, and live cell sorting for further analysis or cloning. For some antigens, labeling cells with fluorochrome-conjugated soluble antigen is a powerful approach (2–4). However, many important antigens are not easily generated in native conformation in soluble form. Conformation can be a critical determinant of epitopes for both anti-virus (5) and autoimmune (6) antibodies. Furthermore, numerous antigenicity-determining features of membrane antigens like glycosylation, interaction with other membrane components, and assembly into multi-subunit complexes such as ion channels depend on expression in the membrane of a suitable cell. Autoantibodies, for example in myasthenia gravis and NMDA receptor encephalitis, bind to complex ion channels whose structures depend on their orientation in the plasma membrane (7). The pathology of Graves' disease is caused by autoantibodies that stimulate the thyrotropin receptor, but studies with monoclonal antibodies suggest that these agonistic antibodies recognize discontinuous, conformation-dependent epitopes, while antibodies that recognize linear epitopes usually do not affect receptor signaling (6). This phenomenon is thought to be the reason why cell-based assays offer superior sensitivity for detection of clinically relevant autoantibodies compared to recombinant protein-based methods like ELISA or immunoprecipitation assays (8).

Our previous studies of the capture of membrane proteins by antigen-specific B cells (9) suggested an approach that would solve several of the problems inherent in assessing B cell specificity for membrane antigens. When a B cell encounters its cognate antigen expressed in the membrane of another cell, it first binds to and then extracts the antigen. This process was first described by Batista et al. (10), and has since been studied in molecular detail (11). During the interaction, the B cell internalizes large quantities of antigen and rapidly becomes highly activated. If the antigen is rendered fluorescent, this enables highly specific sorting of the antigen-specific B cells. The first advantage of this system is that it enables the use of antigens in their native conformation and natural cellular environment. The second advantage is that because antigen capture leads to activation of the B cell, markers such as CD69 can be used to distinguish between a B cell that has internalized antigen and a B cell that is bound by the antigen for some other reason. The third advantage is that adherent cells can be used as antigen donors, and after antigen-specific B cells have contacted their target antigen and bound the donor cells with high avidity, the majority of non-specific cells can be washed away.

We developed this approach using transgenic mouse B cells of known specificity, and then used it to identify, phenotype and clone human peripheral blood B cells specific for the influenza protein hemagglutinin (HA), and the autoantigen acetylcholine receptor (AChR). Hemagglutinin was chosen as a clinically relevant, viral membrane antigen, B cells specific for which are relatively abundant in the blood of vaccinated donors. Hemagglutinin-binding B cells can be labeled with fluorescent soluble antigen, enabling us to compare the efficiency of the new technique with an established method. The complex membrane protein AChR was chosen as a clinically important autoantigen, B cells specific for which are present in the blood of patients suffering from myasthenia gravis, but are rare and difficult to isolate with available methods.

## Materials and Methods

### Mice and Primary Immune Cells

C57Bl/6 mice were bred in the University of Basel Mouse Core Facility. FluBI mice were bred from founders provided by Hidde Ploegh and Stephanie Dougan (Whitehead Institute, Cambridge, Mass). IgH MOG mice (12) were bred from founder members provided by Guru Krishnamoorthy and Hartmut Wekerle, Max-Planck-Institut für Neurobiologie, Martinsried, Germany.Primary immune cells were obtained from spleens by mechanical disruption followed by brief settlement under gravity to remove tissue fragments. B cells were obtained by negative selection using Pan B Cell Isolation Kit II (Miltenyi, cat 130-104-443). All procedures involving animals were authorized by the Cantonal Animal Research Commission.

### Human Samples

Healthy donors between 25 and 65 years old gave written informed consent according to procedures reviewed by the institutional ethics committee (49/06). Some were vaccinated with the 2013, 2014, 2015, or 2016 seasonal influenza vaccine Agrippal^®^, containing inactivated influenza virus surface antigens (hemagglutinin and neuraminidase) from type A/H1N1 (A/California/07/2009). Blood was drawn into S-Monovette tubes (Sarstedt, 7.5ml K3E, REF 01.1605.100, 1.6 mg EDTA/ml blood) before the vaccination and 7–14 days after vaccination, as specified in figure legends. Peripheral Blood Mononuclear Cells (PBMC) were separated over Ficoll-Paque (Axon Lab, Switzerland) according to the manufacturer's instructions and frozen in 1 ml FCS-10% DMSO (FCS from Gibco, DMSO from Sigma Aldrich). Blood for serum was drawn into S-Monovette tubes containing clotting activator (Sarstedt, 7.5 ml Z, REF 01.1601.100) and left at room temperature for 30–60 min, before centrifuging at 2,000 g for 10 min at room temperature. Serum was aliquoted and frozen at −80°C. B cells were isolated from frozen PBMC by rapid thawing in 10 ml pre-warmed complete RPMI medium, incubation for 1 h at 37°C, centrifugation and resuspension in ice-cold separation buffer, followed by negative isolation with magnetic beads from Miltenyi (human B cell Isolation Kit II, cat no. 130-091-151). This kit includes anti-CD43 among the negative selection antibodies, and therefore depletes plasmablasts. Yields of B cells varied from 2 to 8% of total PBMC depending on the donor.

### Plasmids and Cell Lines

A fragment encoding amino acids 1–529 (Genbank ACP41105.1) was amplified from VG11055-C encoding influenza A/California/04/2009 hemagglutinin (Sino Biological, Beijing, China), and fused to an oligonucleotide (Microsynth, Switzerland) encoding amino acids 530–566. The mutation tyrosine-to-phenylalanine (Y98F) in the sialic acid binding site of hemagglutinin (HA) was incorporated by template switching PCR and cloned into the PigLIC expression vector, which confers puromycin (Gibco) resistance. To make the plasmid encoding the fusion protein HA-Y98F-GFP, we amplified GFP from pcDNA6.2C-EmGFP-DEST (Invitrogen) and fused it to the mutated HA construct described above between amino acid 566 and the STOP codon. MOG-mCherry expressing cells were prepared by stably transfecting TE671 cells with a plasmid made by inserting the N-terminal 204 amino acids of rat myelin oligodendrocyte glycoprotein into the cloning site of pcDNA3 mCherry LIC cloning vector (a gift from Scott Gradia, Addgene plasmid # 30125).

TE671 rhabdomyosarcoma cells (referred to as “TE cells” throughout the text, and as “TE 0” when not transfected with additional antigens) were from ATCC (LGC, Wesel, Germany). TE cells were cultured in complete RPMI medium (10% heat-inactivated fetal calf serum (FCS), 100 units/ml of penicillin and 100 ug/ml of streptomycin; all from Gibco), at 37μC in 5% carbon dioxide. TE671 cells were chosen because they grow adherently in a monolayer, are easily transfectable, and being of a muscle cell type, support the expression of the multi-subutnit acetylcholine receptor (AChR). TE cells were transfected with the HA-Y98F-GFP construct and selected with puromycin. Positive transfectants were identified by GFP fluorescence and extracellular immunolabeling against A/California/07/2009 hemagglutinin and sorted to yield the TE CA09HA-GFP cell line. Predicted intracellular location of the GFP moiety and extracellular location of HA were verified by protease sensitivity assay, as follows: TE0 cells and TE-HA-GFP cells were trypsinized, washed three times with PBS, resuspended in HBSS with 5 mM CaCl, and incubated with or without Pronase (Sigma Aldrich, 2 mg/ml) for 4 h at 37°C. Cells were then washed and incubated with human anti-HA IgG primary antibody then PE-conjugated goat anti-human IgG (Jackson ImmunoResearch, 109-116-098) for 30 min on ice and resuspended in PBS. Fluorescence in GFP and PE channels was measured on a CytoFLEX flow cytometer (Beckman Coulter) and results are shown in Supplementary Figure 1. Cells were tested for mycoplasma infection (LookOut Mycoplasma PCR Detection Kit, Sigma Aldrich). Mouse fibroblasts transfected with human CD40 Ligand (Edgar Meinl, Ludwig-Maximilians-Universität, Munich, Germany), used as feeder cells for EBV transformation, were cultured in complete DMEM medium supplemented with 0.5 mg/ml of G418 Sulfate (cat no. 10131-035, Gibco). For irradiation, cells were washed in PBS, trypsinized, resuspended in ice-cold FCS and kept on ice during irradiation (75 Gy).

### Live Cell Imaging

TE671 cells stably transfected with GFP-fused hemagglutinin (from influenza A/WSN/1933) were plated in 8-well chambered coverslips (Ibidi cat no. 80826) and allowed to adhere overnight in an incubator at 37°C in 5% carbon dioxide. The next day, B cells isolated from a FluBI mouse were labeled with Lysotracker Deep Red (Thermo Fisher) according to the manufacturer's instructions, washed and kept in complete RPMI on ice. The chambered coverslip was put in a temperature-, CO_2_-, and humidity-controlled chamber (INU-TIZ-F1 controller, Tokai Hit) into a Nikon A1R confocal microscope with a 60x, 1.40 NA oil immersion objective. The pinhole was opened to 5.0 Airy units and laser power, PMT voltages, and voxel dimensions were optimized to minimize laser light exposure. One stack of confocal sections per minute was captured, and stacks were assembled into frames with Nikon Elements software.

### FACS Isolation of Antigen-Specific B Cells

B cells were isolated from PBMC after influenza vaccination, co-cultured for 3 h with CTV-labeled TE CA09HA-GFP cells, retrieved and incubated with PerCP-Cy5.5-conjugated anti-human CD19 (for IgG ELISpot experiments) diluted 1:20 in cold separation buffer (PBS 2% FCS, 1 mM EDTA) or with APC-conjugated anti-human CD45 (for EBV transformation experiments and high-throughput B cell activation) diluted 1:50 in cold separation buffer and sorted into Eppendorf tubes (FACSAria III Cell Sorter, BD Biosciences). Cells were gated on scatter to select live, single cells; then in two ways to exclude antigen-donor TE cells: CTV negative, and CD19 or CD45 positive. From these putative single, viable B cells, subgates were used for sorting “GFP-capturing,” i.e., GFP-positive, and “GFP-non-capturing,” i.e., GFP-negative. Non-specific surface membrane labeling of adherent cells by bioorthogonal click chemistry was achieved by incubating the cells overnight with 50 μM L-azidohomoalanine in methionine free medium, then washing and incubating for 1 h with 5 μM A647-tagged DIBO-derivative in HBSS at 37°C. The procedure for isolating AChR-binding B cells was similar, but stable transfection with the HA-GFP antigen was replaced by transient transfection with the multi-subunit AChR, including a GFP-variant of the alpha subunit described by Leite et al. (13). Transient transfection resulted in AChR expression by about half the cells, and for subsequent screening of antibody binding to AChR, we always compared binding to the transfected vs. untransfected cells to normalize for antigen-independent binding. Also, for sorting AChR-specific B cells, the antigen independent labeling with A647 was omitted, and instead a second positive (i.e., specific antigen-dependent) label was added with alpha-bungarotoxin conjugated to A647.

### ELISpot for Detection of HA-Specific, IgG-Secreting Human B Cells

96-well plates (Human IgG B cell ELISpot kit (Mabtech, Sweden, Code: 3850-2A) were coated overnight at 4°C with hemagglutinin (Sino Biological, Influenza A H1N1 (A/California/04/2009) hemagglutinin (HA) Protein (His Tag), cat no. 11055-V08B) at 5 μg/ml, or anti-IgG capture-antibody at 15 μg/ml to enumerate total IgG-producing cells, or bovine serum albumin at 5 μg/ml to enable assessment of specificity, washed with sterile PBS and blocked with complete RPMI medium. B cells were isolated, co-cultured with TE mHA-GFP, labeled with anti-human CD19, and GFP-capturing and non-capturing CD19-positive B cells were sorted as described above into coated plates containing 200 μl/well complete RPMI medium supplemented with 1 μg/ml R848 and 10 ng/ml recombinant human IL-2. After culturing for 3 days, the cells were discarded, the plates were washed five times with PBS, and developed by incubating with biotinylated anti-human IgG, followed by streptavidin-AP and BCIP/NBT substrate solution to visualize IgG spots. Antibodies, IL-2, R848, and solutions were provided with the kit and all steps followed the Mabtech protocol. Plates were imaged and read by AID ELISpot reader (software version 7.0, build 14790, AID GmbH, Strassberg, Germany). Results are shown as number of counted spots.

### EBV Transformation of FACS-Isolated Hemagglutinin-Specific B Cells

GFP-capturing and non-capturing B cells were sorted into 1.5 ml Eppendorf tubes containing 200 μl complete RPMI medium, mixed gently with 500 μl of pre-warmed EBV supernatant (ATCC-VR-1492 Epstein-Barr virus, strain B95-8, used neat) and incubated for 1 h at 37°C. Flat-bottomed 96WP were prepared containing 30,000 irradiated CD40L mouse fibroblasts per well in RPMI medium containing 20% non-heat-inactivated FCS, 100 units/ml of penicillin, 100 μg/ml of streptomycin and 1 μg/ml of R848 (Mabtech, Sweden, REF 3611-5X), referred to as “RPMI-20” throughout the text. B cells were added to plates at 30 cells per well and cultured for at least 2 weeks. Proteins, Antibodies and Vital Dyes

Bovine serum albumin (cat no. A4503) was obtained from Sigma Aldrich. Influenza A H1N1 hemagglutinin (A/California/04/2009) protein (cat no. 11055-V08B) and rabbit monoclonal anti-HA antibody RM10 (cat no. 11055-RM10) were obtained from Sino Biological. PerCP-Cy5.5 anti-human CD19 (clone HIB19, BD Biosciences, cat no. 561295), BV510 anti-human CD20 (clone 2H7, BD Biosciences, cat no. 563067), APC anti-human CD45 (clone HI30, BD Pharmingen, cat no. 555485), PerCP-Cy5.5 anti-mouse CD69 (clone H1.2F3, Biolegend, cat no. 104521), APC-Cy7 anti-mouse B220 (clone RA3-6B2, BD Biosciences, cat no. 552094), PE anti-human IgG (Jackson Immunoresearch, cat no. 109-116-098), Alexa Fluor 488 anti-human IgM (Jackson Immunoresearch, cat no. 109-545-129), PE anti-rabbit IgG (Jackson Immunoresearch, cat no. 111-116-144). Anti-human IgG/HRP (cat no. P0214) and anti-human IgM/HRP (cat no. P0215) both obtained from Dako. Cell Trace Violet was obtained from Thermo Fisher Scientific (cat no. C34557) and DAPI from Sigma Aldrich. BV421 anti-human CD27 (clone M-T271, BD Horizon, cat. 562513), BV510 anti-human CD20 (clone 2H7, BD Horizon, cat. 563067), BV605 anti-human IgM (clone G20-127, BD Horizon, cat. 562977), BV711 anti-human CD21 (clone B-ly4, BD Horizon, cat. 563163), PE anti-human CD69 (clone FN50, Biolegend, cat. 310906), PE CF594 anti-human CD138 (clone MI15, BD Horizon, cat. 564606), PE-Cy7 anti-human IgD (clone IA6-2, BD Pharmingen, cat. 561314), PerCP-Cy5.5 anti-human CD19 (clone H1B19, BD Pharmingen, cat. 561295), Alexa Fluor 700 anti-human IgG (clone G18-145, BD Pharmingen, cat. 561296), APC-eFluor 780 anti-human CD38 (clone HIT2, eBioscience, cat. 47-0389-42), APC-Cy7 anti-human CD11c (clone Bu15, Biolegend, cat. 337217), BUV395 anti-human CD71 (clone M-A712, BD Biosciences, cat. 743308).

### Phenotyping of Human Peripheral Blood B Cells

PBMC samples collected before and 7 days after influenza vaccination from each of 9 donors were thawed, and B cells isolated by negative magnetic isolation. TE CA09HA-GFP cells were incubated overnight with 50 μM L-azidohomoalanine in methionine free medium, then washed and incubated for 1 h with 5 μM A647 tagged DIBO-derivative in HBSS at 37°C. B cells were co-cultured with these cells for 3 h, retrieved, and incubated with either an antibody panel containing anti-human CD138 (donors 1–5), or an antibody panel lacking anti-human CD138 and containing anti-human CD11c and anti-human CD71 (donors 6–9) for 20 min on ice. B cells were then washed and acquired on a LSRFortessa cytometer (BD Biosciences) configured with five excitation lasers (355, 405, 488, 561, 640 nm) and 20 detectable parameters. Data in.fcs format were exported from the FACSDIVA operating software of the cytometer and either processed directly using FlowJo (version 10.1, FlowJo, LLC) or re-exported and read into R using the flowCore package (14), and clustered with the k-means algorithm. Heatmaps were generated with the heatmap algorithm of base R. Gating strategies for flow cytometry experiments are shown in Supplementary Figure 3.

### Flow Cytometric Antibody Assay

One hundred microliter of flow buffer containing 50,000 each of unlabeled TE mHA and CTV-labeled TE 0 cells were mixed and incubated with 25 μl of supernatant from EBV transformed B cell clones for 30 min on ice, washed three times with cold flow buffer, labeled with PE-conjugated anti-human IgG and Alexa Fluor 488-conjugated anti-human IgM for 20 min on ice, washed twice with cold flow buffer and measured by flow cytometry. A similar technique was used to measure anti-AChR antibodies in sera and culture supernatants, but using TE cells transiently transfected with AChR-GFP. A647-conjugated α-bungarotoxin (1 μg/ml Thermo Fisher cat. B35450) was used as a positive control.

### ELISA

Bovine serum albumin and Tween were from Sigma Aldrich, PBS from Gibco, TMB for chromogenic development from KPL (SureBlue RESERVE, TMB Microwell Peroxidase, 53-00-00). 96 well-plates (Corning Costar 3590 96well EIA/RIA plate flat bottom without lid) were coated with hemagglutinin and BSA, each at 5 μg/ml, overnight at 4°C with shaking, then washed three times with PBS-0.05% Tween and blocked with PBS-2% BSA at room temperature for 2 h with shaking. Supernatants from FACS-isolated, EBV-transformed, putatively hemagglutinin-specific B cell clones, and from GFP-non-capturing, putatively non-hemagglutinin-specific, negative control B cell clones, were diluted 1:3 in PBS-0.5%BSA. Plates were incubated with diluted supernatants for 2 h at room temperature with shaking, washed three times with PBS-0.05% Tween and incubated with rabbit anti-Human IgG HRP (1:6,000) or rabbit anti-human IgM HRP (1:1,000) in PBS-0.5% BSA for 1 h at room temperature with shaking. Plates were washed three times with 250 μl/well PBS-0.05% Tween and developed with TMB until a blue color was visible. The reaction was stopped with 1N HCl and the plates read at 450 nm immediately after stopping.

### Hemagglutination Inhibition and Virus Neutralization Assays

The titer of influenza A/California/04/09 (H1N1) antibody in B cell culture supernatant samples was measured by HI assay according to the World Health Organization (WHO) protocol manual on animal Influenza diagnosis and surveillance (WHO/CDS/CSR/NCS/2002.5 Rev. 1), following our previously described procedure (15). The neat supernatant samples were pre-treated with 3-fold of cholera filtrate (cat no. C8772-1VL, Sigma-Aldrich) overnight at 37°C to remove non-specific inhibitors. The samples were 2-fold serial diluted in V-shaped 96-well microtiter plate (cat no. 3897, Corning Costar) with PBS. Twenty-five microliter of corresponding influenza antigen A/California (H1N1) antigen (4 HA units) (cat no. 14/134, NIBSC) was added to each well. After 30 min incubation, 50 μl of 1% of chicken erythrocytes (cat no. CLC8800, Cedarlane) was added to each well for 30 min. The antibody titer was measured by tilting the plate based on erythrocyte agglutination and non-agglutination reactions. The positive serum and back titration controls were included in the assay plate.

For neutralization assays, supernatants were incubated with live influenza A/California/2009 virus at various dilutions and then the pre-incubated virus was added to susceptible MDCK cells. After 16 h at 37°C, the cells were fixed and productive infection was detected with an anti-influenza nucleoprotein primary antibody, an enzyme-conjugated secondary antibody, and a colorigenic substrate and the optical density at 450 nm measured by spectrometry. Values <0.15 were considered to indicate viral inhibition.

### High-Throughput B Cell *in vitro* Expansion and ELISA

High-throughput B cell activation and supernatant screening by ELISA (16) followed the method published by Huang et al. (17). FACS-isolated, GFP-capturing and non-capturing B cells were plated at approximately 1.6 cells per well into 384 well plates containing IL-2, IL-21, and irradiated mouse CD40L cells to induce activation and expansion of the B cells. After 12 days, supernatants from these B cell clones were assayed as described above (see section ELISA) with the addition of tetanus toxoid, anti-IgM, and anti-IgG capture antibody-coated wells. cDNA encoding heavy and light chains were cloned from 35 GFP-capturing B cell cultures producing anti-HA antibodies, and expressed recombinantly using standard methods. In later experiments, we replaced the irradiated PBMC with irradiated TE671 cells stably expressing CD40L, and omitted the IL-2.

### Assessment of Specificity and Sensitivity of GFP-Antigen Capture by Transgenic B Cells

Wild type and FluBI mouse B cells were isolated using mouse CD19 microbeads from Miltenyi (cat no. 130-052-201). FluBI B cells were labeled with cell trace violet, diluted with unlabeled wild type B cells at 1:100, 1:1,000 and 1:10,000 and co-cultured for 2.5 h with TE cells expressing HA-GFP. B cells were retrieved, labeled with anti-B220 and anti-CD69 antibodies and subjected to flow cytometry. The population of putatively antigen-specific, i.e., CD69-high and GFP-high cells was then examined for CTV labeling to determine the numbers of true and false positives and negatives, and thus the sensitivity and specificity of the technique. The effect of extracellular antigen quenching used a similar experiment, but with a 1 h co-culture time, no CD69-labeling, and flow cytometric measurements of GFP acquisition in the presence or absence of 0.1% trypan blue. The influence of membrane stiffness was assessed with a similar experiment, with the addition of a pretreatment step exposing the antigen-expressing cells to 0, 1, 3, or 10 μM mycalolide B (AG Scientific, San Diego, California), followed by washing with medium before adding the B cells. The proportion of internalized antigen was studied by comparing immunolabeling following fixation and permeabilization of the B cells, or fixation without permeabilization. After retrieving from the co-culture, B cells were fixed in 4% paraformaldehyde for 15 min at room temperature, then permeabilized in 0.1% saponin in PBS. HA was detected with a rabbit polyclonal antibody (Sino Biological, Beijing, China; 11692-T54) and an A657-conjugated goat polyclonal secondary (Jackson 111-605-003).

### Statistics

Statistical treatments are specified in each figure legend. We used GraphPad PRISM 6 and various algorithms in R/Bioconductor to graph and analyze the data. Numerical results that passed appropriate tests of normality were analyzed by analysis of variance, and otherwise by appropriate non-parametric tests.

## Results

### Membrane Antigen Capture Enables Identification of Mouse and Human Hemagglutinin-Specific B Cells

**Figure 1:**
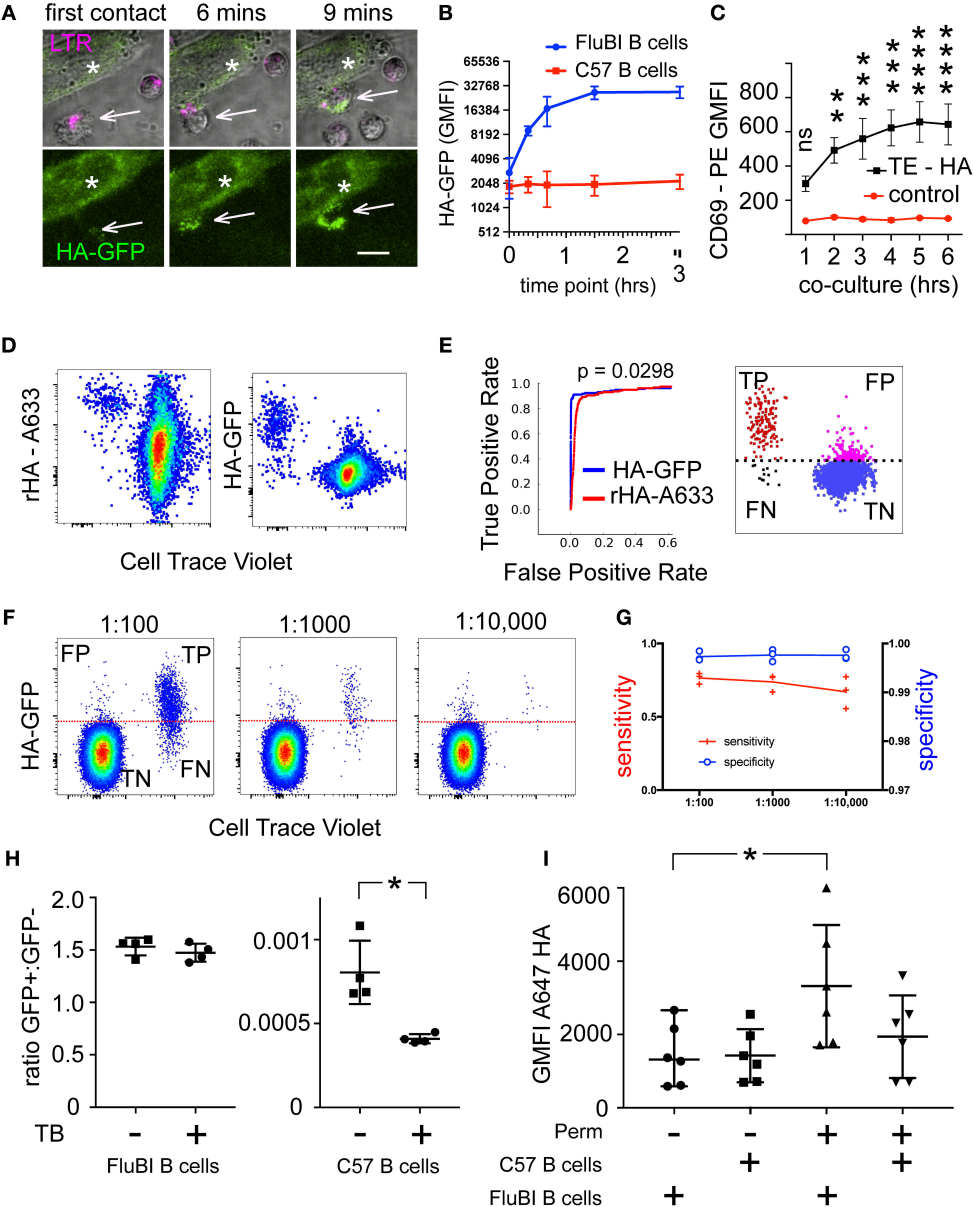
Identification of Hemagglutinin-Specific Transgenic Mouse B Cells by Capture of Membrane-Expressed HA-GFP. **(A)** Live Cell Imaging of membrane antigen capture. Hemagglutinin-specific FluBI B cells (white arrows) labeled with Lysotracker Deep Red (LTR, magenta) are added to cells (white asterisks) expressing a fusion protein comprising the transmembrane and antigenic extracellular domains of hemagglutinin, with a cytoplasmic GFP moiety (HA-GFP, green). Extracellular location of the HA epitope, and the intracellular location of GFP were confirmed by examining sensitivity to extracellular protease (Supplementary Figure 1). Images show the initial contact between a B cell and an antigenic target cell, then the same location at 6 and 9 min later. The upper three micrographs show both fluorescent channels merged with a transmitted light channel to show the morphology of the cells (single confocal planes). The lower panels show only the GFP channel (maximum intensity projections) to show capture of antigen (scale bar =10 um). **(B)** Extraction of membrane-expressed HA-GFP by antigen-specific or antigen-irrelevant B cells. HA-specific FluBI B cells were labeled with Cell Trace Violet (CTV), mixed with unlabeled, antigen-irrelevant, C57 mouse B cells at a ratio of 1:10, and added to an adherent layer of TE671 cells stably transfected with membrane-expressed HA-GFP. At the indicated time points, the B cells were retrieved, immunolabeled with anti-B220 antibody, and interrogated by flow cytometry. The FluBI cells were separated from the C57 B cells by CTV label, and GFP levels were compared between the two cell types. Points and bars show mean and standard deviation of the geometric mean GFP fluorescence. **(C)** Time-course of CD69 upregulation. HA-specific FluBI mouse B cells were exposed to HA-expressing TE HA, or HA-non-expressing TE cells (TE 0) for the indicated times, and then retrieved, immunolabeled for B220 and CD69 and measured by flow cytometry. Vertical axis shows the geometric mean immunofluorescence intensity and SEM of the anti-CD69 signal on B cells exposed to TE HA (black lines and squares), or TE 0 (red lines and circles). Asterisks show significant difference between TE 0 and TE HA conditions at each time point (two-way ANOVA, followed by Sidak's multiple comparison test, (ns = not significant, ^**^p < 0.01, ^***^p < 0.001, ^****^p < 0.0001). **(D)** Comparison of techniques for labeling antigen-specific B cells. CTV-labeled, polyclonal C57 mouse B cells were spiked with 1% unlabeled HA-specific FluBI B cells, and the mixture subjected either to labeling with Alexa-633-labeled soluble hemagglutinin (left), or to co-culture with adherent TE HA-GFP cells (right). Dot plots show flow cytometric measurements of CTV on the horizontal axis (CTV-negative, HA-specific FluBI cells appear to the left of the C57 cells), and the antigen labels on the vertical axes. The frequency of B cells recognizing this hemagglutinin is more than 90% in FluBI mice, but < 1 per 100,000 among polyclonal B cells from naïve, wild-type C57 mice (18), implying that the majority of the wild-type C57 B cells that bind antigen in this context can be considered false positives, whose binding is BCR-independent. **(E)** Receiver Operating Characteristic (ROC) Curves comparing the two methods shown in **(D)**. For three experiments like **(D)**, true- and false positive rates were calculated for each method at various thresholds. The red curve shows the resulting ROC curve for the soluble hemagglutinin label and the blue curve for the membrane-expressed hemagglutinin-GFP fusion protein. Areas under the curves were calculated for each method for three experiments, and compared by two-tailed, paired *t*-test. The schematic on the right shows the four populations, true positives (TP, red), false positives (FP, magenta), false negatives (FN, black), and true negatives (TN, blue) at a given threshold (example threshold shown here by a broken black line). **(F)** Discriminating antigen-specific cells using membrane capture at different target cell frequencies. 10^6^ unlabeled C57 mouse B cells were spiked with 1, 0.1, or 0.01% of CTV-labeled HA-specific FluBI B cells [note that the CTV labels the spike in this paradigm, opposite to the paradigm shown in **(D)**], and the mixture co-cultured with adherent TE HA-GFP cells. A threshold was set at approximately 0.1% false positives, the FluBI and C57 cells were distinguished using the CTV label. Then, we calculated the sensitivity [true positives/(true positives + false negatives)] and specificity [true negatives/(true negatives + false positives)]. **(G)** Performance of antigen-capture labeling at different target cell frequencies. Data from three experiments like **(E)** are plotted on two vertical axes, at the target cell frequencies shown on the horizontal axis. Specificity is plotted with open blue circles on a blue line on the right axis (0.97–1.0), and sensitivity is plotted with red crosses on a red line on the left axis (0.0–1.0). **(H)** Effect of extracellular fluorphore quenching on apparent antigen signal. After co-culture with TE HA-GFP cells, FluBI B cells (left column scatter graph) or wild-type C57 B cells (right column scatter graph) were measured by flow cytometry in the presence or absence of 0.1% trypan blue. The vertical axis shows the ratio of GFP+ cells to GFP negative cells. Asterisk shows significant difference between trypan blue treatment conditions (^*^p < 0.05, unpaired, two-tailed *t*-test). Pooled data from two independent experiments. **(I)** Effect of permeabilization on antigen immunodetection. After co-culture with TE HA-GFP cells, FluBI B cells (1st and 3rd columns) or wild-type C57 B cells (2nd and 4th columns) were fixed, and either permeabilized with saponin or not, before immunolabeling with a rabbit anti-HA antibody and an A647-conjugated anti-rabbit secondary antibody. The vertical axis shows the flow cytometric geometric mean fluorescence intensity of the secondary antibody. Asterisk shows significant difference between permeabilization conditions within the FluBI condition (^*^p < 0.05, unpaired, two-tailed *t*-test). Pooled data from three independent experiments.

The phenomenon of membrane antigen capture by B cells is illustrated by the live cell imaging sequence in Figure 1A. Upon contacting cognate antigen expressed in the membrane of another cell, B cells rapidly extract and internalize large quantities of antigen. In the experiment shown, B cells from FluBI mice (18), which are specific for influenza hemagglutinin (HA), were exposed to adherent cells expressing hemagglutinin fused to GFP (TE HA-GFP cells).

We measured the time course of HA-GFP uptake by antigen-specific FluBI and antigen-irrelevant C57Bl/6 B cells. Hemagglutinin-specific B cells avidly extracted the HA-GFP fusion protein, with GFP uptake reaching a maximum between 45 and 90 min, while antigen-irrelevant B cells capture almost no GFP (Figure 1B). We further expected that CD69 expression after exposure to cognate antigen-expressing adherent cells would be time-dependent (9). We confirmed this in FluBI mouse B cells, and also showed that CD69 induction depends on expression of the antigen. The total duration of the co-culture is also important; CD69 expression increases with longer exposure (Figure 1C).

To explore the possibility of exploiting this phenomenon for identifying antigen-specific B cells from among a polyclonal population, we spiked polyclonal mouse B cells from wild type C57Bl/6 mice with varying numbers of FluBI B cells. The two kinds of B cells were pre-labeled before mixing, to enable their separation later. To compare the performance of the antigen capture method with the soluble fluorescent antigen labeling method, one sample was co-cultured with an adherent layer of TE HA-GFP cells and the second sample was labeled with fluorochrome-conjugated recombinant HA. Both methods resulted in sensitive detection of the hemagglutinin-specific FluBI B cells (Figure 1D). To make a more quantitative comparison, we extracted a Receiver Operating Characteristics (ROC) curve from the results of three such experiments and compared the curves obtained by labeling with fluorescent soluble antigen or by antigen extraction (Figure 1E). The antigen-extraction method performed significantly better than the soluble fluorescent antigen method, with comparable sensitivity but better specificity. To assess the performance of the antigen-capture technique at physiologically realistic frequencies of antigen-specific B cells, we repeated the experiment with serial dilutions of FluBI B cells in wild type C57Bl/6 B cells (Figure 1F) and detected HA-specific B cells down to a frequency of 1/10,000. This is in the range of naturally occurring influenza-specific B cells in humans (19). Specificity was always above 99%, and sensitivity varied between 55 and 80% (Figure 1G).

We observed a small number of HA-GFP-positive wild type B cells, for example in Figures 1D,F. We hypothesized that these are false positives, generated by superficial association of donor cell debris with the B cell membrane surface. To test this, we compared the signal in the presence and absence of trypan blue, which has been reported to quench fluorophores with the spectral characteristics of GFP (20). We reasoned that antigen internalized by the BCR-dependent pathway would be physically separated from the quencher and therefore protected from the quenching effect. As shown in Figure 1H, the GFP signal on wild type B cells was approximately halved by quenching, while the signal from FluBI cells was unaffected.

To confirm that the increase in GFP fluorescence in FluBI B cells was due to internalized antigen, rather than superficially membrane-associated debris, we compared the intensity of immunofluorescence after immunolabeling the captured antigen with an anti-HA antibody in permeabilized or unpermeabilized cells. Results are shown in Figure 1I. Immunofluorescence was significantly increased after permeabilization in FluBI but not in wild type B cells.

The supposition that the GFP-positive cells among the wild type C57 B cells are antigen-non-specific also predicts that a similar number would be seen among FluBI and C57 B cells if the fluorescent antigen were non-cognate for both cell types. We tested this prediction using myelin oligodendrocyte glycoprotein (MOG) fused to GFP or mCherry as a non-cognate membrane antigen. B cells from IgH MOG BCR transgenic mice do capture this antigen and served as a positive control. The ratio of antigen-acquiring to non-acquiring B cells was consistently 1,000-fold higher for cognate B cells than for antigen-mismatched B cells. There was no significant difference between different mismatched B cell–antigen combinations (Supplementary Figure 4).

Since physical properties of the antigen donor cell membrane, such as stiffness and compliance, have an impact on the capture of antigens in immune complexes from follicular dendritic cells (21), we examined the effect of manipulating membrane stiffness with the actin depolymerizing agent mycalolide B. As shown in Supplementary Figure 5, as membrane stiffness is reduced, acquisition of antigen by non-cognate B cells increases, while cognate antigen capture decreases.

The strong adherence of B cells to other cells expressing their cognate antigen also offers another possibility for isolating antigen-specific B cells in co-culture with adherent antigen-expressing cells. This can be exploited by washing off non-binding B cells after a short period of co-culture. We examined the effect of the length of the pre-wash co-culture (Supplementary Figure 2A), and discovered that a time of about 20 min is optimal. Using this technique, which we call “panning,” significant increases in efficiency can be achieved (Supplementary Figures 2B,C).

**Figure 2:**
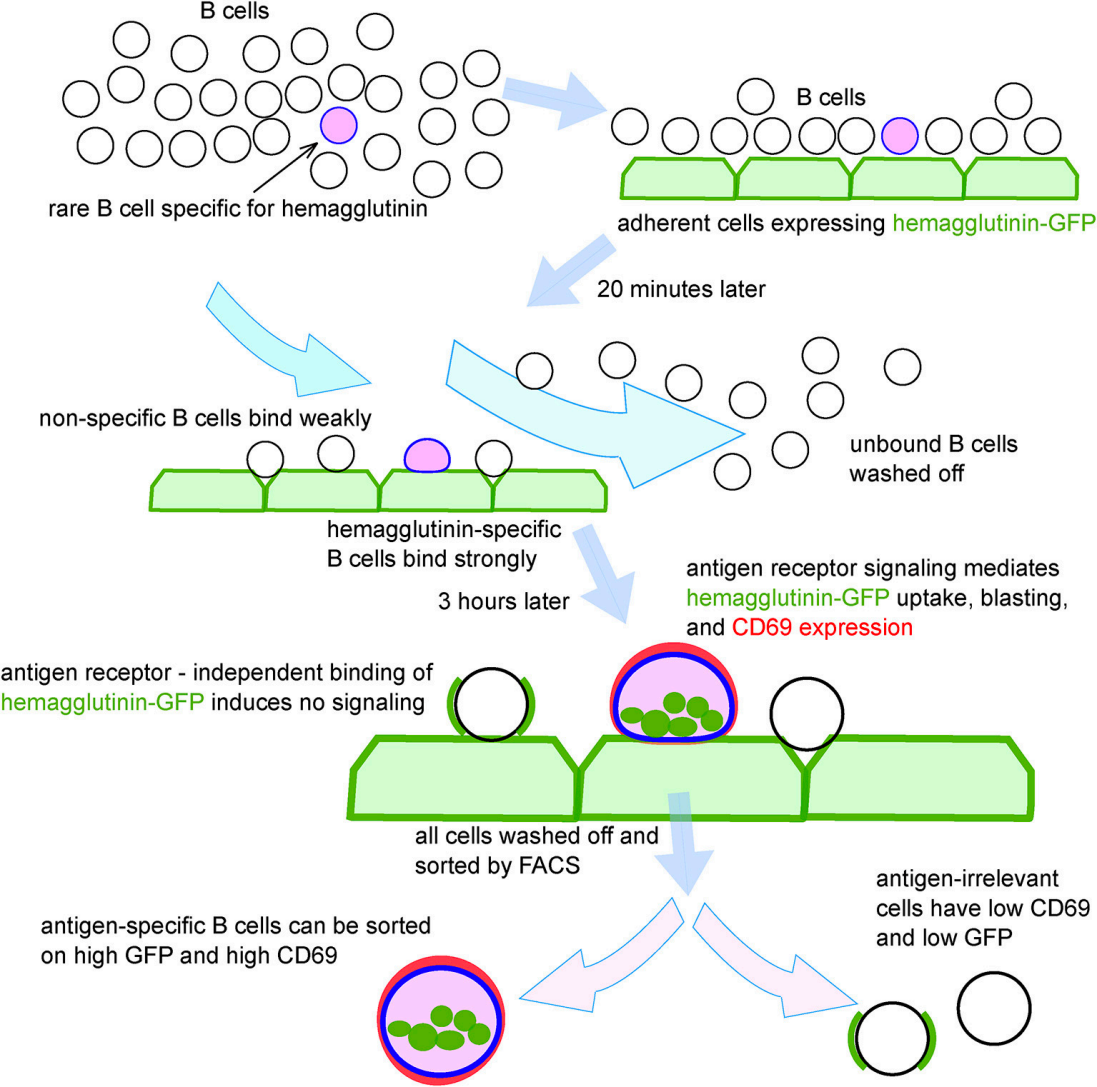
Schematic depiction of process for isolation antigen-specific B cells using adhesion to antigen-expressing cells, fluorescent antigen capture, upregulation of B cell activation markers, and fluorescence-activated cell sorting.

**Figure 3:**
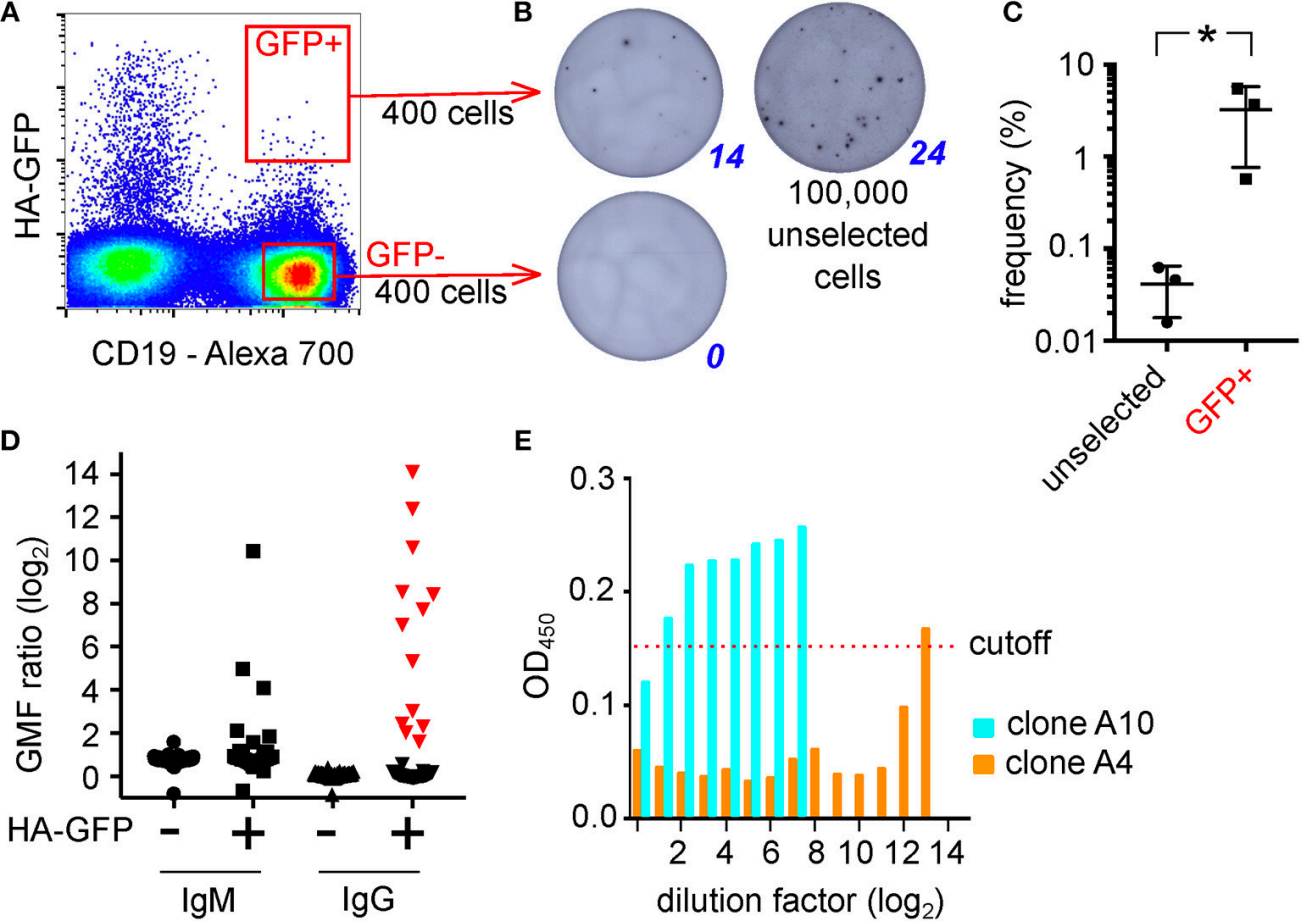
Isolation of Human Hemagglutinin-Specific B Cells by Capture of Membrane-Expressed HA-GFP. **(A)** Gating of HA-GFP-capturing B cells. Polyclonal human B cells were cultured for 3 h on an adherent layer of TE HA-GFP cells, then retrieved and labeled with an anti-CD19 antibody and the CD19-positive, GFP-high cells were sorted by FACS. **(B)** Enumeration of HA-specific antibody-secreting cells by ELIspot. 400 GFP-capturing B cells sorted as shown in **(A)**, or the same number of GFP-negative cells, or 100,000 unsorted polyclonal B cells from the same donor were incubated in ELIspot wells coated with hemagglutinin for 3 days, then the spots of antibody were visualized with anti-human IgG secondary antibody. **(C)** Enrichment of antigen-specific B cells by membrane antigen capture. Three experiments like the one described in **(B)** were completed, using B cells from donors 2 weeks after immunization, and for each the frequencies of HA-specific IgG-producing cells among the GFP-capturing population and the unsorted population were calculated and compared by two-tailed paired *t*-test. The mean frequency was enriched by ~100-fold from 0.04 to 3% (^*^p < 0.05). **(D)** EBV-immortalized clones derived from HA-GFP-capturing B cells. GFP-capturing or non-capturing B cells were sorted as in **(C)**, immortalized with Epstein Barr Virus (EBV), and cultured for 4 weeks. The supernatants were screened by flow cytometry for anti-HA IgM and IgG. Vertical axis shows the log ratio of immunofluorescence on HA-expressing cells divided by the same value for non-expressing control cells: i.e., a value of 0 implies no specific binding. Supernatants plotted with red triangles in the 4th column (HA-specific IgG-containing supernatants clones derived from GFP-capturing B cells) were screened for hemagglutination inhibition and virus neutralization. **(E)** Neutralization of influenza virus by supernatants from EBV clones. Supernatants from the clones shown with red triangles in **(D)** were incubated with live influenza A/California/2009 virus at the indicated dilutions and then the virus was added to MDCK cells. After 16 h, the cells were fixed and productive infection detected by immuno-colorimetry. Vertical axis shows optical density at 450 nm, and values <0.15 imply viral inhibition. Here, clone A10 exhibits minimal neutralization, and clone A4 shows neutralization down to a dilution factor of 212 (1:4,096, or 50 ng/ml).

The steps of the technique, as optimized using transgenic mouse B cells, and model antigens, are shown schematically in Figure 2. To test the system in the context of a natural immune response, we exposed B cells from human peripheral blood mononuclear cells (PBMC) to adherent TE671 cells stably expressing a GFP-tagged version of the hemagglutinin from influenza A/California/2009, the H1N1 strain included in influenza vaccines from 2010 until 2016 (TE CA09HA-GFP). We introduced the point mutation Y98F in the hemagglutinin to eliminate sialic acid mediated binding (22). After 3 h of co-culture, B cells were retrieved and the GFP-capturing B cells were isolated by FACS (Figure 3A). We assessed the antigen-specificity of the sorted cells by anti-HA IgG ELIspot (Figure 3B). Comparing unselected B cells and HA-GFP-capturing B cells from the same donor, the technique enriches the hemagglutinin-specific B cells by approximately 100-fold (Figure 3C). To obtain clones for characterization of the secreted antibodies, the experiment was repeated, and 5,000 GFP-high, CD45-positive cells, and a similar number of GFP-non-capturing cells were transformed with Epstein Barr Virus (EBV). Four weeks later, the culture supernatants were assayed for HA-binding activity by ELISA and flow cytometry. Out of 46 clones derived from HA-GFP-capturing B cells, 13 produced HA-specific IgG as measured by flow cytometry, and 1 produced hemagglutinin-specific IgM (Figure 3D). None of the supernatants from 49 non-GFP-capturing clones bound specifically to hemagglutinin. To verify that hemagglutinin binding measured by these assays corresponds to antigen-specific immunoglobulin binding, we assayed IgG from the 13 hemagglutinin-binding supernatants for hemagglutination inhibition and virus neutralization. Three clones showed neutralizing activity, of which one showed strong virus-neutralizing activity (Figure 3E), and also hemagglutination inhibition, confirming the potential of the technique to select and identify B cells of relevant affinity and specificity. We also examined the effect of panning on the efficiency of isolation of human influenza-specific B cells, and showed that more than 80% of sorted B cells secrete HA-specific antibodies in subsequent culture (Supplementary Figures 2B,C).

### *In vitro* Single Cell B Cell Cultures of Sorted Cells for Immunoglobulin Cloning

**Figure 4:**
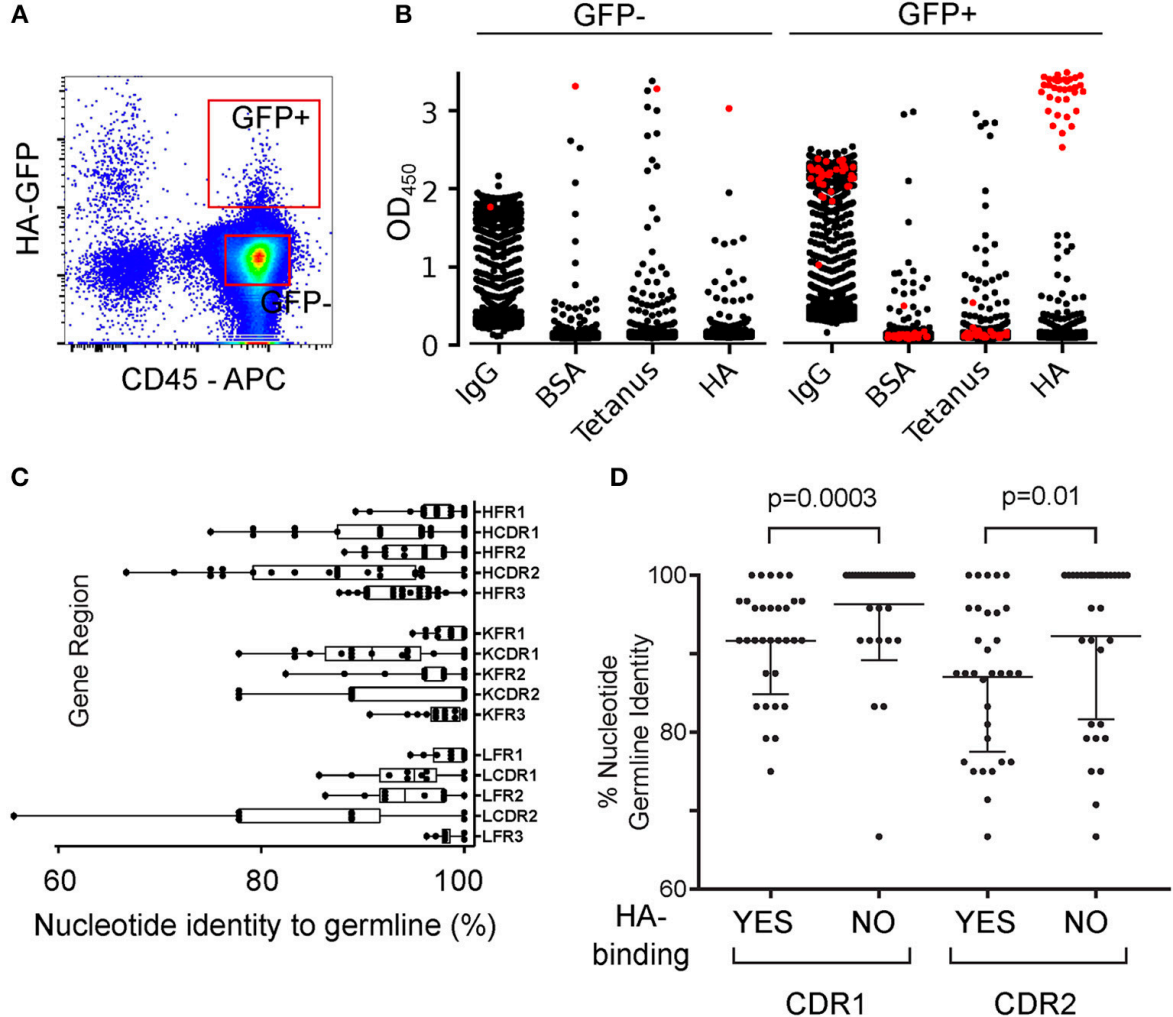
Single Cell Cloning and Immunoglobulin Sequencing of Hemagglutinin-Extracting Human B Cells. **(A)** Gating of HA-GFP-capturing B cells. Human B cells were cultured for 3 h on an adherent layer of TE HA-GFP cells, then retrieved and labeled. CD45-positive, GFP-high cells were sorted by FACS and dispensed into wells of 384-well plates at 1.6 cells per well. **(B)** Specificities of IgG in supernatants from high-throughput B cell cultures. GFP-high and GFP-negative B cells were sorted and distributed into wells as in **(A)**, and expanded for 18 days with irradiated PBMC and cytokines. The supernatants were then screened by ELISA for total IgM, total IgG, and binding to hemagglutinin (HA), tetanus toxoid (Tetanus), or Bovine Serum Albumin (BSA). Vertical axes show the optical density at 450 nm. Red symbols correspond to supernatants binding to HA, of which there are 34 derived from GFP-high cells and one from a GFP-negative cell. IgG from the single GFP-negative cell-derived clone also bound to other antigens and was deemed non-specific. Raw numbers, and ELISA optical densities of IgG-producing, and HA-specific IgG-producing clones are shown in Supplementary Figure 6. **(C)** Distribution of mutations in the immunoglobulin genes of hemagglutinin-capturing B cells. RNA from the cultures described in **(B)** was reverse-transcribed and segments corresponding to variable regions of heavy, lambda, and kappa chains were amplified by PCR. We examined antigen binding of the recovered recombinant antibodies to confirm the correct pairing of heavy and light chains, and compared sequences of the cloned gene segments with the closest germline sequences from publicly available databases. Germline conservation is indicated by the percentage of nucleotide identity to the nearest-match V gene segment for each of the germline-encoded structural components: FR1, framework 1; CDR1, complementarity-determining region 1; FR2, framework 2; CDR2, complementarity-determining region 2; FR3, framework 3. Amino acid sequences and inferred V, D, and J gene segments are shown in Supplementary Table 1. **(D)** Comparison of somatic hypermutation in heavy chain immunoglobulin sequences isolated from HA-enriched (HA-GFP-capturing) and HA-binding (in first-round screen) hits vs. hemagglutinin-non-binding control sequences. Hemagglutinin-specific VH sequences (*n* = 32) represent antibodies that were verified to bind HA after cloning and re-expression. HA-non-binding VH sequences (*n* = 33) represent heavy chains that were cloned from wells containing HA-binding antibodies, but did not contribute to a hemagglutinin-binding heavy-light chain pair in the validation screen. *p*-values from Mann-Whitney test. Germline identity was assessed with IgBLAST using the IMGT germline database.

The EBV cloning immortalization efficiency was too low to give informative coverage of immunoglobulin genes of the antigen-extracting population, so we adopted the protocol for expansion of single human B cells described by Huang et al. (17). HA-GFP-capturing and non-capturing B cells from a donor 2 months after immunization (Figure 4A) were put into 384-well plates containing irradiated PBMC, anti-CD40, IL-2 and IL-21 to induce proliferation and plasma cell differentiation. Antibodies in the supernatants of these B cell cultures were measured by ELISA for total IgM, total IgG, and specific IgM or IgG against hemagglutinin (Figure 4B). From 1920 supernatants of HA-GFP-capturing B cell cultures and 1920 from non-capturing controls 39.6% of the GFP-capturing B cells (761 supernatants) and 38.9% of the non-capturing B cells (747 supernatants) produced IgG (Supplementary Figure 6). Thirty-five of the supernatants from GFP-capturing B cells and none of the supernatants from GFP-non-capturing cells bound specifically to hemagglutinin. From those 35 wells (which initially contained 1.6 cells per well on average), 27 recombinant antibodies were recovered which bound specifically to hemagglutinin. Hemagglutinin binding of some of the antibodies is dependent on both the heavy and light chains, and in some cases only on the heavy chain (Supplementary Figure 7), as has been reported previously ((23–25)). The sequences had a significant number of mutations from germline V gene segment sequences, with mutations enriched in the complementarity-determining regions (Figure 4C). This suggests that the B cells, which captured the antigen *ex vivo*, had an antigen-experienced history, had received T-cell help, and undergone affinity maturation. In parallel, we examined the mutation rate in 33 HA-non-binding heavy chains cloned from the same donor, and the number of clones with unmutated immunoglobulin genes was significantly higher than in the hemagglutinin-capturing cells (Figure 4D). All of the 27 heavy and light chain pairs of the HA-binding antibodies were unique. This suggests that the number of available hemagglutinin-specific clones is at least at the higher end of the serum antibody clonotypic diversity, which has been estimated to be between 50 and 400 clones (26, 27). However, we found an over-representation (5/27, 19%) of antibodies with the combination of VH1-18 and VK2-30. According to DeKosky et al. (28), VH1-18/VK2-30 pairings comprise <0.1% of total clones identified for any of the donors, suggesting that in the donor we examined, the VH1-18/VK2-30 pair has some germline-encoded affinity for hemagglutinin.

### A Second, Antigen-Independent Label Reports BCR-Independent Binding

**Figure 5:**
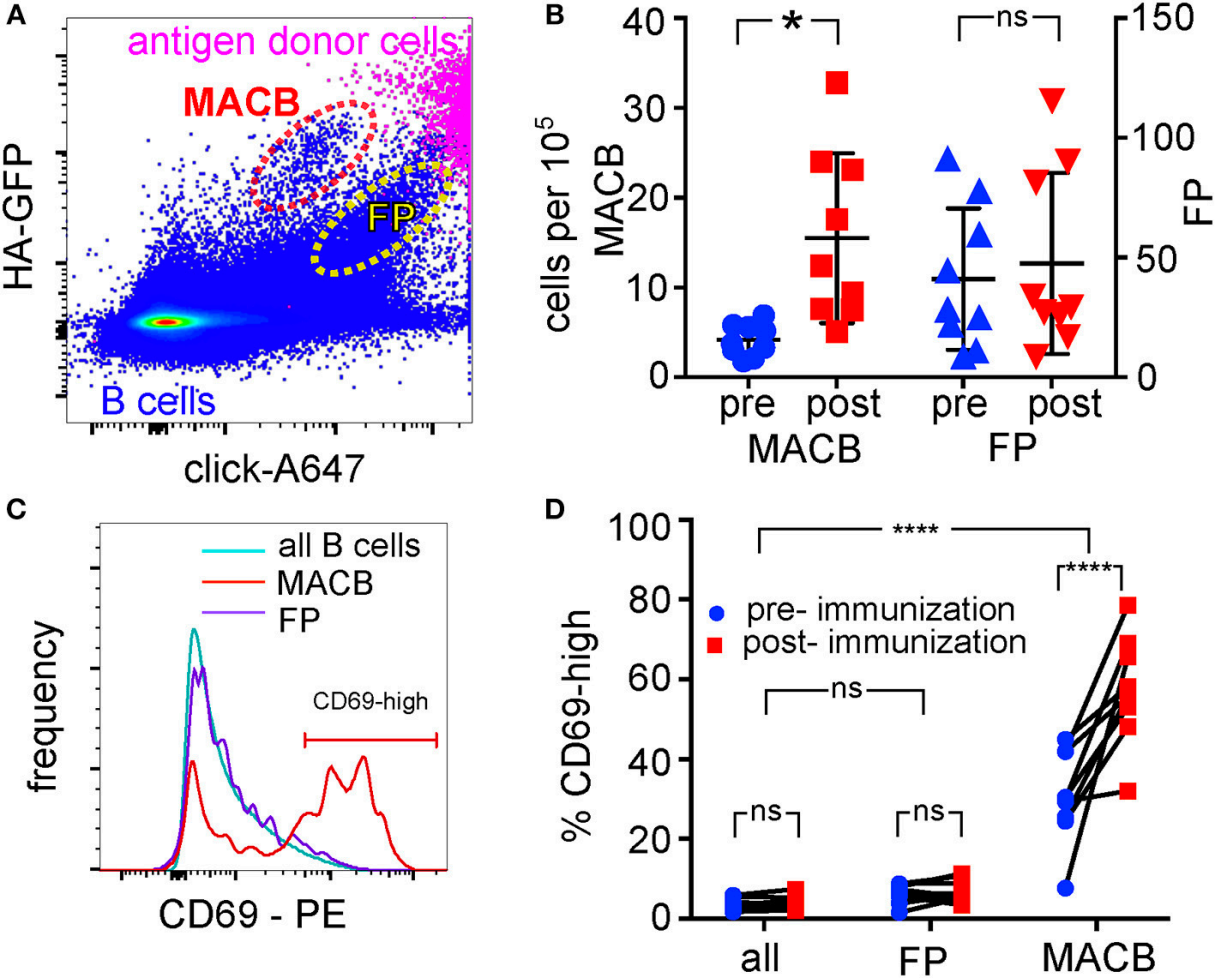
Identification of Antigen-Specific Population with Antigen-Associated and Antigen-Independent Labels. **(A)** BCR-dependent and non-specific antigen uptake. B cells were co-cultured for 3 h with TE HA-GFP cells, of which the extracellular domains of membrane proteins had been additionally labeled with Alexa 647 (A647) by click chemistry. B cells were then retrieved and immunolabeled for CD19, measured by flow cytometry, and gated on single B cells. The plot shows GFP intensity plotted against A647 intensity, both for the B cell population (blue pseudocolor, lower half of the figure), and, for comparison, the antigen-expressing TE cells (magenta, upper right corner). Most B cells capture neither HA-GFP, nor other proteins (bottom left corner of plot). Two populations of B cells acquire HA-GFP: those that acquire high GFP and intermediate A647 (“MACB”–membrane antigen-capturing B cells, broken red oval); and those that also acquire large quantities of A647 (labeled “FP”–false positive, broken yellow oval). Gating is shown in Supplementary Figure 3. **(B)** Increase in numbers of HA-GFP capturing cells after immunization. B cells from nine donors were collected before and 1 week after influenza immunization, and then all 18 samples were assayed as above. Numbers of cells falling in the MACB gate (1st and 2nd scatter columns, left vertical axis), and the FP gate (3rd and 4th scatter columns, right vertical axis) shown in **(A)** were compared before and after immunization for each donor by two-way ANOVA followed by Sidak's test. Vertical axes show numbers of cells in the gates per 100,000 B cells measured. Bars show means and standard deviations ^*^p < 0.05. **(C)** Histograms of CD69 expression by the cells in the three populations. CD69 expression on cells in populations MACB, FP, and the global B cell pool was measured by flow cytometry. The horizontal bar on the right of the plot marked “CD69-high” shows the gate used to determine the percentages shown in **(D)**, and also in analyses hereafter of the “CD69-high” sub-population of antigen-extracting cells. **(D)** CD69 expression, and response to immunization by the cells in the three populations. Samples from nine donors taken before and after immunization were measured and gated as in **(C)**, and the levels of CD69 compared between populations and between time points by two-way ANOVA, followed by Dunnett's test to compare the two populations with the whole B cell pool, and Sidak's test to compare pre- vs. post-immunization within each population (^****^p < 0.0001).

Sorting the HA-GFP-capturing cells enriched hemagglutinin-specific B cells by a factor of 100. However, the sorted population still contained 90% of B cells not specific for hemagglutinin. We hypothesized that this is due BCR-independent binding of antigen-donor cell fragments to irrelevant B cells, and that this non-specific signal could be distinguished by adding a second, antigen-independent membrane label. To test this hypothesis, we labeled exposed membrane proteins on the TE CA09HA-GFP antigen-donor cells with Alexa Fluor 647 (A647) using bioorthogonal click chemistry before co-culturing with human B cells. We reasoned that BCR-mediated capture of cognate antigen would result in the uptake of a large amount of antigen-GFP, and a small amount of antigen-associated A647, proportional to the GFP signal. BCR-independent mechanisms of uptake, such as adhesion of donor-cell-derived vesicles, would result in a higher ratio of A647 to GFP. As is clear from Figure 5A, both kinds of events indeed occur–there is one GFP-high, A647-intermediate population that we hypothesize are membrane antigen-capturing B cells (MACB); and one population with a lower GFP:A647 ratio similar to the donor cells. We tested our hypothesis that antigen-specific cells would be contained in the GFP-high, A647 intermediate population in two ways: by tracking the size of the populations before and after influenza immunization, and by examining the activation of this population after exposure to antigen.

B cells from 9 donors, from blood drawn before or 1 week after influenza vaccination, were co-cultured with A647-labeled TE CA09HA-GFP cells for 3 h, then retrieved and immunolabeled for flow cytometry. In all 9 donors, the number of cells in the MACB population increased following immunization, while no consistent increase or decrease was seen in the false positive population (Figure 5B).

We also compared the expression of CD69 in the two populations of B cells, as well as the global expression level of CD69 expression. The false positive population had levels of CD69 indistinguishable from the global population, while the MACB population showed a bimodal distribution, with a lower peak like the global population and a CD69-high peak (Figure 5C). The MACB population was the only population with the second peak, and the only one whose CD69 expression was influenced by immunization (Figure 5D). We concluded that the combination of these three directly antigen-capture-related markers is sufficient to identify antigen-extracting B cells.

### Phenotypic Characterization of Influenza-Specific B Cells

**Figure 6:**
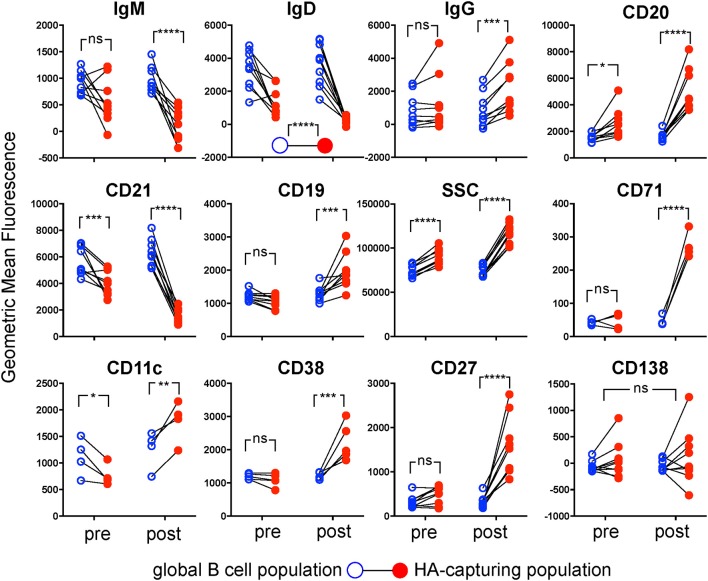
Phenotypes of Hemagglutinin-Specific B Cells from Peripheral Blood. For the nine donors shown in Figure 5D, expression of markers was compared between MACB cells (solid red circles) and the whole B cell population (open blue circles), before (left on each plot) and after (right on each plot) immunization, by two-way repeated measures ANOVA. Paired samples from each donor are linked by black lines. Four donors were assessed with an antibody panel including CD11c and CD71, and 5 donors assessed with a panel including CD38 instead. For all parameters shown except IgD and CD138 there was a significant interaction between population and time-point, so the antigen-extracting population was compared to the whole population at the two time-points independently with Sidak's test (^*^p < 0.05, ^**^p < 0.01, ^***^p < 0.001, ^****^p < 0.0001).

**Figure 7:**
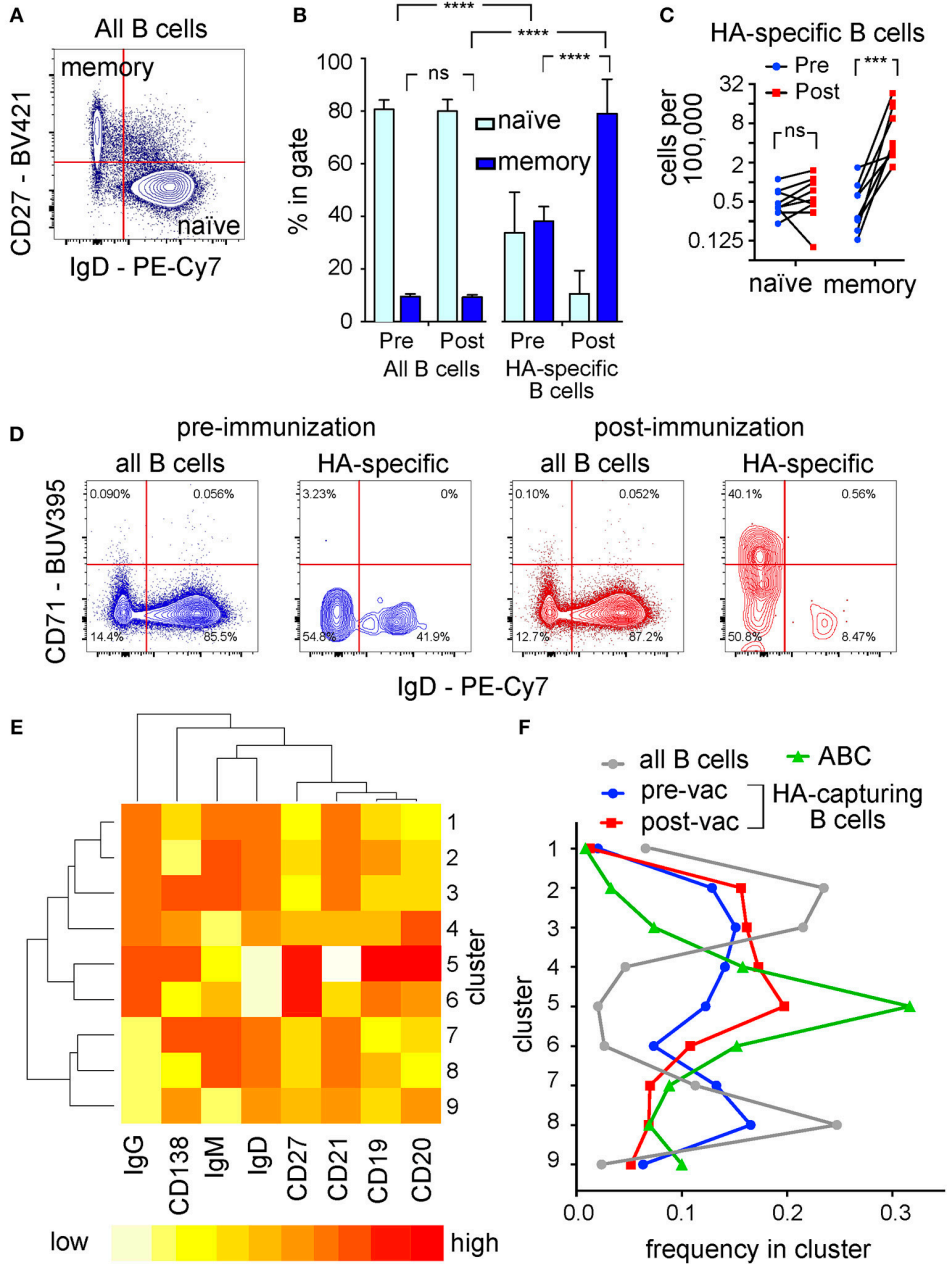
Antigen Experience Profiles of Hemagglutinin-Specific B Cells Detected in Human Blood. **(A)** Representative dot plot of IgD vs. CD27 expression. Human peripheral blood B cells were co-cultured and analyzed by flow cytometry as described in Figure 5. The plot shows IgD immunofluorescence on the horizontal axis and CD27 immunofluorescence on the vertical axis, for the whole B cell population. Percentages of events in the “memory” and “naïve” quadrants shown were used to generate **(B)**, and absolute numbers of cells in these quadrants were used to generate **(C)**. **(B)** Proportions of events in the naïve (light blue bars) and memory (dark blue bars) quadrants in samples taken before and after immunization. Left half of bar chart shows proportions of naïve and memory cells in the whole B cell pool; right half shows proportions among the hemagglutinin-specific fraction. In each sub-chart, the two bars on the left correspond to samples taken before immunization, and the two bars on the right to samples taken after. Following two-way, repeated measures ANOVA, the differences between frequencies of memory cells before and after immunization were subjected to Sidak's test; and the differences between frequencies of memory cells in hemagglutinin-specific vs. the total B cell population to Dunnett's test (^****^p < 0.0001). **(C)** Absolute numbers of naïve and memory cells in the hemagglutinin-specific population. Numbers of cells corresponding to the “memory” phenotype [upper left quadrant in **(A)**], and “naïve” phenotype [lower right quadrant in **(A)**] in the hemagglutinin-specific population per 100,000 total B cells are plotted on a log scale. Pre-immunization values are plotted with blue circles, and post-immunization with red squares, and paired samples from each donor are linked by black lines. Following significant two-way ANOVA, the values were compared between the two time points within the two populations by Sidak's test (^***^p < 0.001). **(D)** Contour plots of IgD vs. CD71 expression. Plots show either the whole B cell pool (1st and 3rd plots), or the CD69-high, hemagglutinin-capturing cells (2nd and 4th plots), taken from one donor, either before vaccination (blue contour plots on left), or 1 week after (red contour plots on right) vaccination. Cells from nine donors falling in the top left quadrants (IgD-negative, CD71-high) of plots corresponding to the 4th plot here were used as the “Activated B Cells” in **(E,F)**. **(E)** Heat map showing the characteristics of 9 phenotypic clusters. Flow cytometry results for the 8 markers shown, for cells taken before and after vaccination from 9 donors were subjected to automated clustering using the k-means algorithm in R/Bioconductor. Dendrogram to the left of the plot shows the hierarchical relationships between the clusters, and the dendrogram above the plot the relationships between the markers. Red color encodes the highest level of expression, and yellow-to-white the lowest. **(F)** Fractions of different cell populations falling in the clusters shown in **(E)**. Gray circles (connected by gray line) show the distribution of all B cells among the clusters. Blue circles show the fractions of hemagglutinin-capturing, CD69 high (HA-specific) B cells from donors before vaccination, and red squares the corresponding population one week after vaccination. Green triangles show the fractions of vaccination-induced “Activated B Cells,” as defined by the IgD-negative, CD71-high quadrant in the 4th plot of **(D)**. Cluster numbers on the vertical axis correspond to cluster numbers shown in **(E)**. Fractions shown on the horizontal axis were calculated by dividing the total number of cells (pooled from all 9 donors) in a given cell population, in a given cluster, by the total number of cells in that population.

Having reliable flow cytometric markers of antigen specificity allowed us to characterize the hemagglutinin-specific B cell subset and compare it to the overall population of B cells. A longitudinal follow-up also enabled us to track phenotypic changes of this population induced by vaccination (Figures 6, 7). All examined markers except CD138 were differently expressed in the hemagglutinin-specific B cells compared to the overall population of B cells (Figure 6). IgM, IgD, and CD21 were significantly reduced in the hemagglutinin-specific population, whereas CD11c, CD19, CD20, CD27, CD38, CD71, and IgG were increased. For most of these markers the differences were more pronounced at the timepoint after vaccination (Figure 6). However, higher CD20 and lower CD21 were also seen in the hemagglutinin-specific population before vaccination. The only marker showing opposing trends before and after vaccination was CD11c; before vaccination, CD11c was lower in the hemagglutinin-specific cells than in the overall B cell population, whereas after vaccination it was higher.

We hypothesized that the clearest population differences between hemagglutinin-specific and other B cells seen after immunization were driven by increases in the numbers of recently activated, vaccine-specific B cells, because patterns associated with memory cells (CD27-high, IgG-high, IgD-low) also characterize the post-vaccination, hemagglutinin-specific population. Based on CD27 and IgD expression (Figure 7A), we plotted the numbers of naïve and memory B cells as fractions of the global, or fractions of the hemagglutinin-specific B cell populations (Figure 7B). Before immunization, about 80% of the global B cell population have a naïve phenotype (IgD-positive, CD27-negative), and about 10% have an IgD-negative, CD27-positive memory phenotype. Hemagglutinin-specific B cells before immunization include about 40% each of naïve and memory cells. Following immunization, the proportions in the global B cell pool remain unchanged from before immunization, while the proportion of hemagglutinin-specific cells with the memory phenotype rises to almost 80%. We hypothesize that these changes reflect an expansion of hemagglutinin-specific memory B cells following immunization, rather than a change in phenotype, because the absolute numbers of naïve cells in the hemagglutinin-specific pool are not changed by immunization (Figure 7C).

The increased abundance following vaccination, and the diminished CD21 expression characterizing the post-vaccination hemagglutinin-capturing B cells led us to hypothesize that this population might be related to the vaccination-induced “activated B cells” (ABC) described by Ellebedy et al. (29). The cardinal features of these B cells are minimal IgD, and high CD71 expression. Plotting IgD against CD71 for the global B cell pool, or for the hemagglutinin-capturing B cells, we see that cells with these features are rare (~0.1%) in the global B cell pool, but comprise 40% of the post-vaccination hemagglutinin-capturing B cells (Figure 7D). Before vaccination, most of the hemagglutinin-capturing B cells are CD71-negative. To examine the relationships between the four populations of cells (global pool, ABC, and the pre-, and post-vaccination HA-capturing B cells) quantitatively and without the assumptions of manual gating, we used the automated clustering algorithm k-means to classify B cells into nine clusters, based on their expression of eight markers (Figure 7E). These markers were chosen to avoid the GFP, A647, CD69, and CD71 that were used to define the cell populations. We then assessed the frequencies of the four cell populations in each of the nine clusters. The highest frequency of ABC was seen in the CD19-high, CD20-high, CD21-low cluster (cluster 5 in Figures 7E,F). This cluster also contained the highest frequency of hemagglutinin-capturing B cells post vaccination, corroborating the hypothesis that these cells are related. Across all nine clusters, the frequencies of post-vaccination hemagglutinin-capturing B cells were low in clusters that contained few ABC, and high in clusters enriched for ABC. The cluster (cluster 8 in Figures 7E,F) characterized by low CD20, low IgD, low CD138 and high IgM was unique in containing equally low frequencies of ABC and post-vaccination hemagglutinin-capturing B cells, but a frequency of pre-vaccination hemagglutinin-capturing B cells almost as high as among the global B cell pool (Figure 7F). These results are consistent with the hypothesis that hemagglutinin-specific B cells circulating in peripheral blood at steady state include naïve cells as well as memory cells, and that following vaccination, one or both of these populations gives rise to the activated B cells described by Ellebedy et al. (29).

Since expression of these markers differs between antigen-capturing B cells and the global B cell pool, we examined the possibility that these markers might be enough to identify the antigen-capturing population without the fluorescent antigen marker. In samples taken post vaccination, this does indeed enable more than 100-fold enrichment, but at steady state, the most promising combination of markers only offers about 10-fold enrichment (Supplementary Figure 8).

### Adapting the Method to Isolate Autoantigen-Specific B Cells

**Figure 8:**
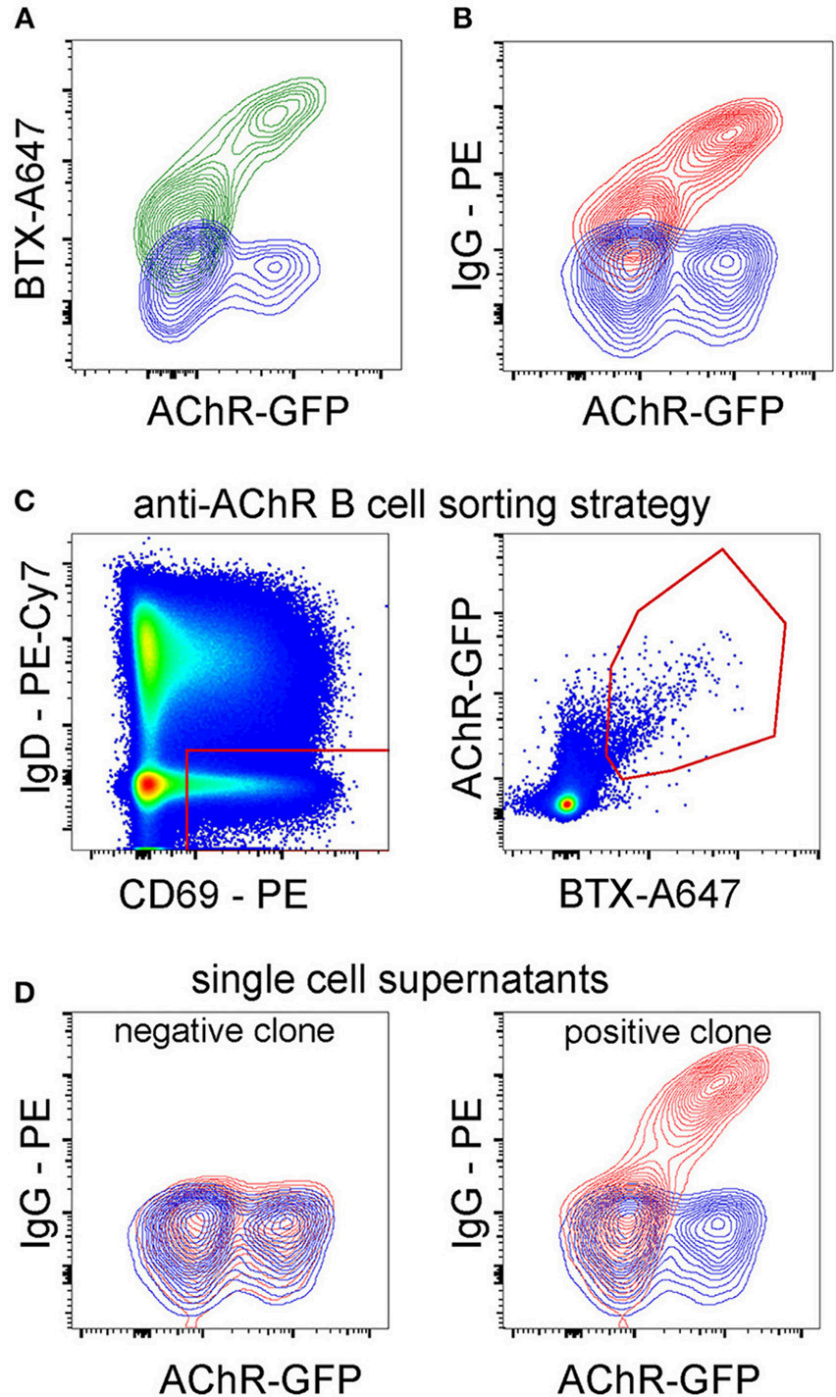
Optimized Sorting Using Membrane-Antigen-Capture Enables Near-Complete Purification of Antigen-Specific Cells. **(A)** A647-labeled alpha-bungarotoxin (BTX) binds to AChR-GFP transfected TE671 proportionally to GFP intensity. TE cells were transiently transfected with the alpha, beta, delta and epsilon subunits of human AChR with a GFP moiety between third and fourth transmembrane helices of the alpha subunit. After 24 h, cells were labeled with A647-alpha-bungarotoxin and analyzed by flow cytometry. **(B)** AChR-binding IgG from patient serum. Serum from a patient diagnosed with myasthenia gravis was diluted to 1:200 and incubated with TE671 cells transiently transfected with AChR-GFP, then with PE anti-human IgG. **(C)** Application of antigen-capture sorting to the isolation of AChR-specific B cells from a human donor. Magnetically isolated peripheral blood B cells from a patient diagnosed with myasthenia gravis were cultured for 3 h with TE cells expressing AChR. The AChR alpha subunit has a GFP moiety fused in-frame in the large cytoplasmic loop between the third and fourth transmembrane helices, and the antigen-donor cells are additionally labeled with A647-conjugated bungarotoxin (BTX). After co-culture, B cells were retrieved by washing, labeled with fluorescent antibodies, and CD69-high, IgD-low, GFP- and A647- double positive cells (gating strategy in Supplementary Figure 3) were sorted. **(D)** Confirmation of AChR-specificity of B cell clone from patient. Single B cells sorted as in C were cultured for 13 days with CD40L-expressing feeder cells and IL-21 as described in Methods. Culture supernatants were then assayed for specific antibodies by incubating with AChR-GFP-transfected (GFP-expressing) or non-transfected (GFP-negative) cells, washing, and detecting with fluorescent secondary antibodies against human IgM, IgA, or IgG. The contour plot on the left shows results from a clone considered negative (similar IgG binding to transfected and untransfected cells), and the plot on the right a positive (higher binding to transfected than untransfected cells) anti-AChR IgG-producing clone.

Having optimized the paradigm using influenza hemagglutinin as a model membrane antigen, we examined its applicability to isolating B cells specific for a more complex membrane protein, for which the fluorescent soluble antigen approach is less suitable. We chose the ligand-gated ion channel nicotinic acetylcholine receptor (AChR), antibodies against which can cause the pathology of myasthenia gravis. The receptor is comprised of five protein subunits, each with four transmembrane domains, and despite recent advances in isolating AChR-specific B cells, they remain a challenging target (30). As antigen-donor cells we used TE671 cells transiently transfected with the alpha, beta, delta and epsilon subunits of human AChR. The alpha subunit was modified by the insertion of GFP in the cytoplasmic loop between its third and fourth transmembrane domains (13) (Figure 8A). Binding of IgG from serum of a patient diagnosed with myasthenia gravis to AChR-transfected cells is shown in Figure 8B. Peripheral blood B cells from this donor were co-cultured for 3 h with AChR-GFP-transfected cells. As an additional specificity marker, transfected cells were labeled with A647-conjugated α-bungarotoxin (a high affinity AChR-binding toxin). B cells were then sorted on scatter, IgD, CD69, and antigen capture, as shown in Figure 8C and Supplementary Figure 3. Single B cells sorted from the antigen-capturing gate were cultivated in 384-well plate wells with IL-21 and feeder cells as described for the culture of HA-specific B cells, and after 13 days, their supernatants were tested for AChR-binding specificity. Examples of negative and positive clones are shown in Figure 8D. Using this technique, the frequency of antigen-specific clones is lower than observed for vaccine-induced hemagglutinin-specific B cells; in this donor, about 0.5% of sorted clones were AChR-binding.

## Discussion

The approach of exposing polyclonal B cells to cell-expressed membrane antigens and then sorting the antigen-capturing cells is a powerful technique for B cell research and antibody engineering. Its advantages include the ability to present antigens in their native state and environment, the possibility to exploit the activation of the B cells themselves to increase specificity, and the gain in efficiency offered by panning the B cells that adhere to the antigenic cell layer. The technique is only appropriate for B cells recognizing integral membrane proteins, but this includes many important antigens including viral glycoproteins, autoantigens, and tumor antigens. We assume that there is a lower limit on the affinity of the BCR-antigen interaction required to enable antigen capture, but for many purposes, the preferential isolation of higher affinity clones is a positive feature.

During optimization of the technique, we cloned and phenotyped hemagglutinin-specific B cells from human peripheral blood. The phenotypic characteristics of antigen-capturing B cells characterized after co-culture can reflect either *in vivo* developmental changes, or changes induced by the capture process. The strong induction of CD69 and the differences in light scattering properties that are seen before and more so after vaccination are probably at least partly a consequence of B cell activation following antigen capture *ex vivo*. We assume that when a significant difference is observed between the hemagglutinin-specific and global B cell populations, following vaccination, but not at steady state, that this is likely to reflect the *in vivo* response to vaccination. Elevated expression of IgG, CD19, CD71, CD38, and CD27 fit this pattern. On the other hand, low IgD, low CD21, and high CD20 appear to be characteristics of hemagglutinin-specific B cells irrespective of recent vaccination. A plausible interpretation of this pattern of results is that a small population of hemagglutinin-specific memory B cells, possibly resulting from previous infections or vaccinations, circulates in blood at steady state. Following vaccination, some of these cells proliferate strongly, and their progeny produce the post-vaccination-typical population we observe, characterized by high CD27, CD38, and CD71 expression. Not all the hemagglutinin-capturing B cells from donors before vaccination had this memory-like phenotype. The membrane-capture technique also identifies a small number of hemagglutinin-specific naïve B cells, and it remains possible that descendants of these cells contribute to the post-vaccination expanded memory pool.

Having developed the method using influenza hemagglutinin as a model antigen, we moved on to AChR, a multi-subunit membrane antigen, and the major autoantigen in myasthenia gravis. The isolation of AChR-specific human B cells remains difficult because the most commonly recognized epitope is dependent on the receptor's native conformation (31). We were able to isolate anti-AChR reactive B cells from 6/6 tested patients. As reported by others (30), the frequency of these B cells was much lower than that of B cells directed against influenza hemagglutinin after vaccination. Because the goal of this project was to obtain patient-derived AChR-specific monoclonal antibodies, sensitivity was a higher priority than specificity. We therefore did not use an antigen-independent label to reject likely false positives, and this may also have depressed our specificity.

These two applications of the technique both involved known target antigens, but the method should be adaptable to isolating B cells that recognize antigens whose identity is not yet known, but that are known to be expressed in the membrane of a particular adherent cell line. For this purpose, membrane components of the antigen-expressing cell line would be chemically labeled with a fluorophore before adding B cells. After co-culture, all B cells that have upregulated CD69 and taken up the fluorophore are sorted and cultured. Antigen-specificity of the secreted antibodies in the B cell culture supernatant can then be tested against the antigen-expressing cell line. Heavy and light chain genes can be cloned from the cultured B cells, and used to prepare monoclonal antibodies for identification of the target antigen by immunoprecipitation and mass spectrometry.

The seminal study of antigen acquisition by B cells from the membranes of other cells by Batista et al. (10) used hen egg lysozyme (HEL) as a model antigen, in one of two forms. Either the antigen was complexed with antibodies and loaded onto an Fcγ receptor-expressing myeloid cell line, or antigen-donor cells were transfected with a construct encoding a transmembrane domain and an extracellular HEL moiety. HEL-specific B cells were seen to form stable contacts or “synapses” with either kind of antigen donor cell, and gather the antigen into the synapse. Since then, evidence has accumulated supporting the idea that B cells in the germinal center acquire antigen in the form of immune complexes (32) from specialized antigen-proffering cells such as follicular dendritic cells (FDC), and studies of the subcellular details of how B cells acquire antigen have focused on this mechanism. The extraction of membrane integral protein antigens demonstrated by Batista et al., has received much less attention, and there are important differences between the two scenarios. In both cases, the ability of the B cell to remove the antigen from the donor cell is dependent on a high enough BCR-antigen binding affinity, but the forces retaining the antigen on the donor cell differ. In the case of FDC-proffered immune complexes, the outcome of the “tug-of-war” between the B cell and the donor cell is dependent on the affinities of several non-covalent interactions between antigen, antibodies, complement components and receptors on the FDC. *In vitro* experiments suggest that when B cells acquire cognate antigen in the form of immune complexes from FDC, they capture the antigen without co-capturing the tethering moiety, i.e., without removing any transmembrane protein from the membrane of the FDC, suggesting that in these circumstances, extraction of the integral membrane protein is energetically less favorable than rupturing one of the protein-protein adhesions in the tethering chain of proteins (21). In the case of an integral membrane antigen, extracting the antigen from the membrane (possibly together with some quantity of the associated membrane) is the only option, unless the antigen is enzymatically cleaved. Enzymatic cleavage is a possibility because B cells can secrete lysosome hydrolases into the synaptic cleft (33), but this mechanism appears to be restricted in its utilization and not to be employed for acquiring antigen from live cells (21). A more analogous physiological equivalent of the mechanism exploited by our technique might be the capture of viral antigen from infected cells, such as lymph node subcapsular macrophages. These cells act as pathogen sentinels, being particularly susceptible to infection with viruses such as vesicular stomatitis virus (34) and are important for early B cell responses against the pathogen (35). The fact that the specificity of the technique is reduced by depolymerizing the actin cytoskeleton of the antigen-donor cells is, however, perhaps analogous to the reduction in affinity discrimination caused by similar treatment of FDC in the immune complex acquisition scenario described by Spillane and Tolar (21). This result also predicts that cells with stiffer membranes will make the most suitable antigen-donors for this technique.

The fluorescent membrane antigen capture method can thus be used in two ways. By using a short *ex vivo* co-culture with antigen donor cells, and using the combination of an antigen-associated fluorophore and an antigen-independent fluorophore to report antigen specificity, very rare populations of antigen-specific B cells can be precisely characterized. By using adherent antigen-donor cells and a long enough co-culture to allow activation and surface expression of CD69, antigen-specific B cells can be efficiently sorted at very high purity and expanded *in vitro* for antibody screening and immunoglobulin gene cloning. In both cases, the technique is powerful enough to detect antigen-specific cells without the need for pre-selection of memory or IgG-positive B cells. A particularly flexible feature of the membrane antigen capture method we describe is that it can be used to detect B cells that recognize an antigen that is expressed on a defined cell type, but whose molecular identity is unknown. This is commonly the situation in the search for anti-cancer antibodies, and autoantibodies involved in autoimmunity, and we envisage that this technique will be applied in these fields to search for new antigens.

## Ethics Statement

This study was carried out in accordance with the recommendations of the Federation of European Laboratory Animal Science Associations. The protocol was approved by the Basel Stadt Cantonal Animal Research Commission.

This study was carried out in accordance with written informed consent from all subjects. All subjects gave written informed consent in accordance with the Declaration of Helsinki. The protocol was approved by the Ethikkommission beider Basel.

## Author Contributions

JL, ET, NS, and TD: conceptualization. MZ, NR, JL, HK, AG, IC, MS, LsK, NS: investigation. AE and LgK: resources. MZ, NR, JL, ET, NS, and TD: writing. RL, LudK, NS, and TD: funding acquisition. RL: supervision.

### Conflict of Interest Statement

The authors declare that the research was conducted in the absence of any commercial or financial relationships that could be construed as a potential conflict of interest.
