# Supplementary material for: Antigen Extraction and B Cell Activation Enable Identification of Rare Membrane Antigen Specific Human B Cells
Source: Front Immunol. 2019 Apr 16;10:829. doi: 10.3389/fimmu.2019.00829 (PMC6477023; doi:10.3389/fimmu.2019.00829)
Supplement: Supplementary file 1 [file Table_1.pdf]

	Heavy Chain				Light Chain		
	**VH**	**DH**	**JH**	**CDR3**	**VK/VL**	**JK/JL**	**CDR3**
1	VH1-18	DH3-10	JH6	ARDRMSPFIWFGATNDYNMDV	VK2-30	JK1	MQGTQWPWT
2	VH1-18	DH3-9	JH4	ARDRRDLLTGPTFDS	VK2-30	JK2	MQGTHRPYT
3	VH3-23	DH3-3	JH4	AKDRVTVVVESGSVDY	VK3-15	JK4	QQYNNWPPLT
4	VH3-30	DH4-11	JH5	ARVHSNYFVGWAGSWFDP	VK1-12	JK4	QQAHSFPLT
5	VH1-18	DH3-9	JH6	ARDRQHTLTGYGMDV	VK2-30	JK2	MQGTHWPVA
6	VH3-15	DH3-10	JH4	TTMGEERLLNVPVVYFHN	VK1-39	JK2	QLGYT
7	VH1-69	DH2-8	JH4	ARSNGSYGGDDY	VK3-11	JK2	QQRSNWLYT
8	VH1-69	DH2-21	JH4	AIDRDSSDY	VK3-11	JK1	QQRGNWLWT
9	VH1-18	DH1-26	JH4	ARDKGNLLSGTFLDY	VK2-30	JK2	MQGTHWPYT
10	VH1-3	DH3-22	JH6	ARARDFLGEAGLIVVAFYGMDV	VK3-11	JK4	QQRSNWPLT
11	VH4-31	DH3-10	JH4	ARDRGLDHLVLGY	VK1D-33	JK4	QQSLT
12	VH3-21	DH6-19	JH4	ARDLPSISVAGPLDY	VK1-17	JK1	LQYNTYPRT
13	VH1-2	DH1-26	JH6	ARGWEQAGLWEAPFRYYYYGMDV	VK2D-29	JK4	MQSRQLPLT
14	VH1-3	DH2-2	JH6	ARAVPAVILGVPWNYGMDV	VK1D-12	JK5	QQTKTFPIT
15	VH1-18	DH1-26	JH5	ARDKGTLLSGSLLDF	VK2-30	JK2	MQGSRWPYT
16	VH3-7	DH6-6	JH4	ARAVIFPNLPIAARPYYFDF	VK1D-33	JK4	QQYADLPLT
17	VH4-4	DH4-17	JH2	AKMDYRDVDLDYWYFDL	VK3-15	JK1	QQYNHWPPL
18	VH4-30-2	DH1-26	JH4	ARADGMTDKLFDY	VL1-40	JL2	QSYDTSLSALV
19	VH4-59	DH6-13	JH5	ARTIATAGTFRFDP	VL3-1	JL2	QTWDSSTVV
20	VH4-4	DH4-17	JH2	ARTDYGDVDLDYWYFDL	VL3-21	JL2	QVWYTSADHPAV
21	VH3-64D	DH6-19	JH4	VKALSKYSSGWQIDY	VL3-21	JL2	QVWDSSTDRPAV
22	VH3-21	DH3-16	JH6	SRDFYDSRGQYYGMDV	VL4-69	JL1	QTWGTGIYV
23	VH4-59	DH6-13	JH5	ARGIAAAGAFRFDP	VL3-1	JL2	QAWDTTTVV
24	VH1-3	DH6-19	JH6	VGPRQWLAAYFYTYGLDV	VL7-43	JL3	LLYIGGTGV
25	VH2-70	DH1-7	JH4	ARTRTTWAFDS	VL1-47	JL2	AAWDDSLNGVV
26	VH4-31	DH6-19	JH4	ARKKAVAGTLWSDY	VL3-1	JL2	QAWDIIAGVV
27	VH3-23D	DH3-10	JH5	AKYAYFHYNWFDP	VL1-40	JL3	QSYDSSLSGSV

Supplementary Table 1. Anti-HA antibody sequence characteristics, including V gene segment usage and the amino acid sequence of the CDR3 resulting from V(D)J recombinationof the heavy and light chains.
