# Supplementary material for: Antigen Extraction and B Cell Activation Enable Identification of Rare Membrane Antigen Specific Human B Cells
Source: Front Immunol. 2019 Apr 16;10:829. doi: 10.3389/fimmu.2019.00829 (PMC6477023; doi:10.3389/fimmu.2019.00829)
Supplement: Supplementary file 2 [file Data_Sheet_1.PDF]

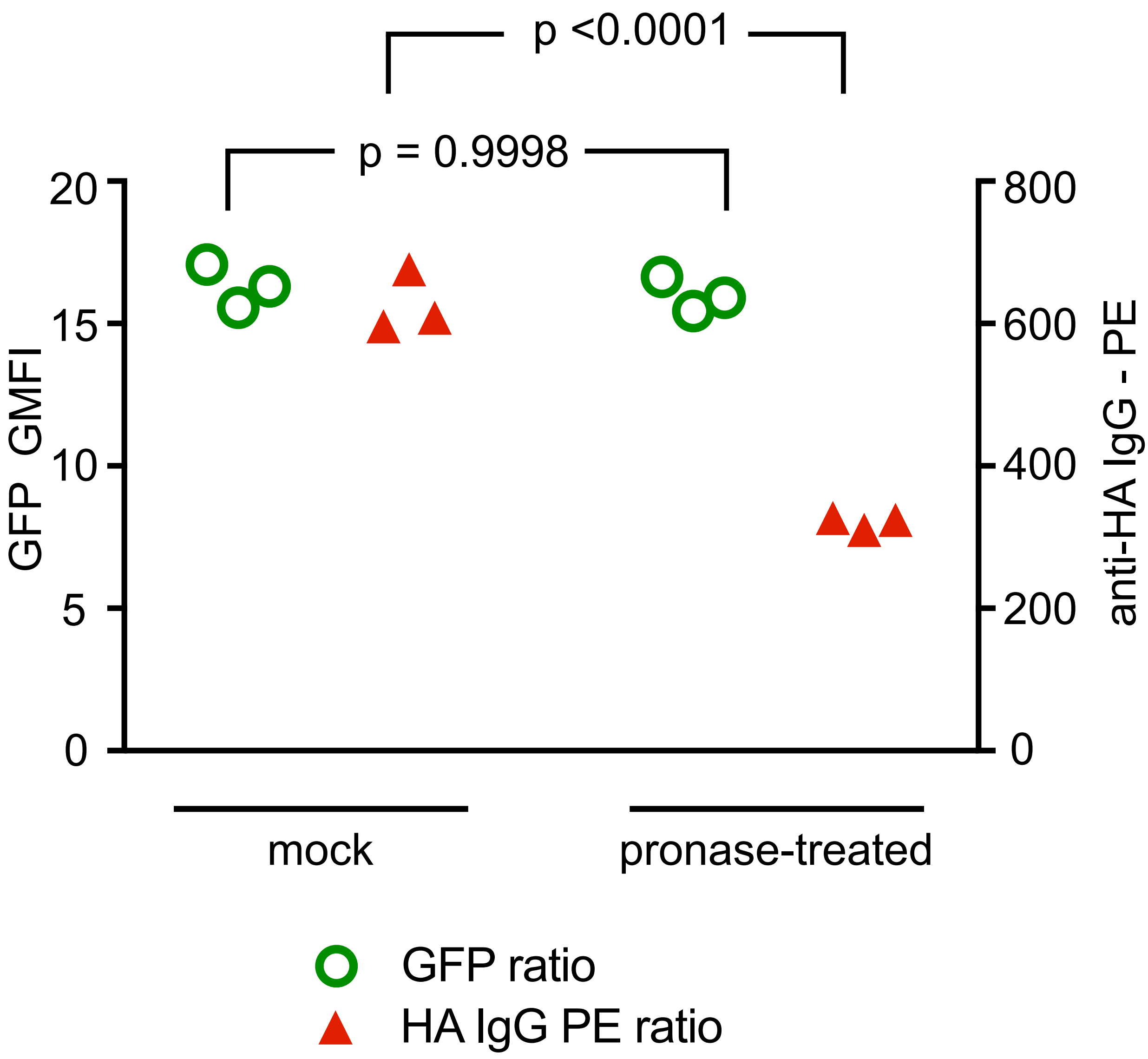


**Supplementary Figure 1.** Sensitivity of Hemagglutinin and GFP to protease treatment. Intact TE cells expressing the fusion protein HA-GFP were treated with pronase for 4 hours at 37°C and then immunolabeled for hemagglutinin. Immunofluorescent intensity and GFP intensity were measured by flow cytometry, and the values for HA-GFP expressing cells divided by the corresponding values for untransfected TE cells. The left vertical axis shows the ratio of GFP fluorescence TE HA-GFP : TE 0 and the right axis shows the ratio for anti-HA immunofluorescence. Points are values from triplicate enzymatic digestions processed independently. Two-way analysis of variance confirmed the presence of an interaction between fluorophore and pronase treatment, and then Sidak's multiple comparison test was used to calculate the p values for the two within-channel comparisons.
