# Supplementary material for: Antigen Extraction and B Cell Activation Enable Identification of Rare Membrane Antigen Specific Human B Cells
Source: Front Immunol. 2019 Apr 16;10:829. doi: 10.3389/fimmu.2019.00829 (PMC6477023; doi:10.3389/fimmu.2019.00829)
Supplement: Supplementary file 3 [file Data_Sheet_2.PDF]

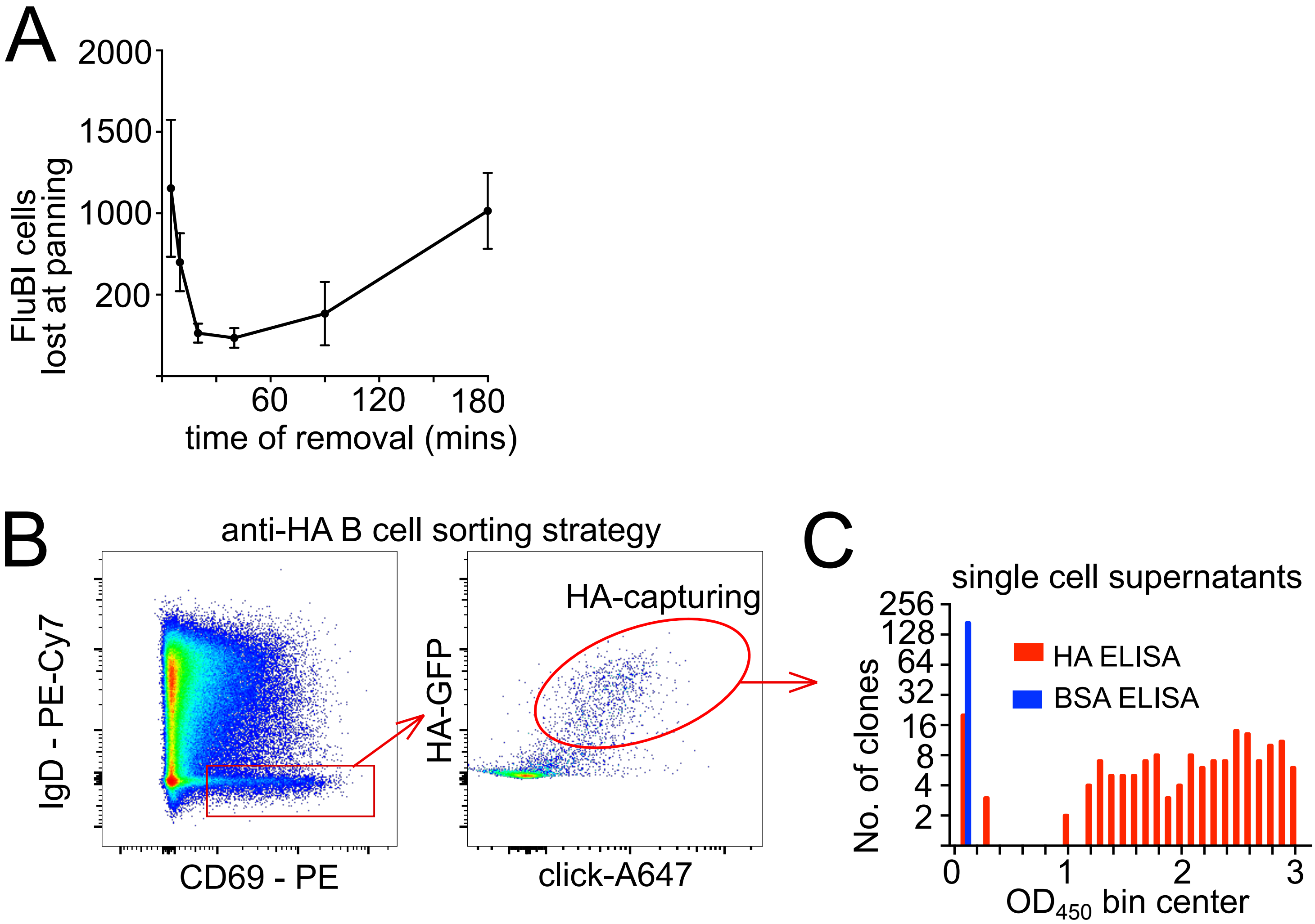

Supplementary Figure 2. Utility of panning to increase efficiency. **(A)** Optimization of panning time. 100,000 polyclonal mouse B cells spiked with 2000 CTV-labeled, HA-specific FluBI B cells were incubated on an adherent layer of HA-GFP-expressing cells for the indicated times, then the supernatant was removed, and the number of FluBI B cells in the supernatant washed off from the adherent cell layer was counted by flow cytometry. Points show the mean and bars show standard deviations of the numbers of FluBI B cells. **(B)** Combining B cell panning, double labeling of antigen, and selecting for IgD-negative and CD69-positive cells enables highly specific B cell sorting. B cells from 3 recently immunized human donors were co-cultured for 25 minutes with A647-click-labeled adherent TE cells expressing HA-GFP. Then, the medium and non-adherent B cells were removed and replaced with fresh medium. 165 minutes later, the remaining B cells were retrieved by vigorous washing, and the CD19-positive, IgD-negative, CD69-high, GFP-high, A647-high population (Supplementary Figure 3) sorted by FACS. The dot plot on the right compares the antigen-associated GFP signal against the ubiquitous surface protein label Alexa 647 on the population of cells sorted as shown in the dot plot on the left. **(C)** HA-specificity of sorted cells. IgD-negative, CD69-high, GFP-high, A647-high cells sorted as shown in (B) were cultivated in 384-well plates for two weeks in the presence of IL-21, IL-2, and CD40L-expressing feeder cells, at a density of 2 B cells per well. Concentrations of total IgG and HA-specific IgG in supernatants from these wells were then assessed by ELISA. Of 168 IgG-producing wells, 146 produced HA-specific IgG, and none produced BSA-binding IgG ($p<0.0001$, Chi squared test).
