# Supplementary material for: Antigen Extraction and B Cell Activation Enable Identification of Rare Membrane Antigen Specific Human B Cells
Source: Front Immunol. 2019 Apr 16;10:829. doi: 10.3389/fimmu.2019.00829 (PMC6477023; doi:10.3389/fimmu.2019.00829)
Supplement: Supplementary file 4 [file Data_Sheet_3.PDF]

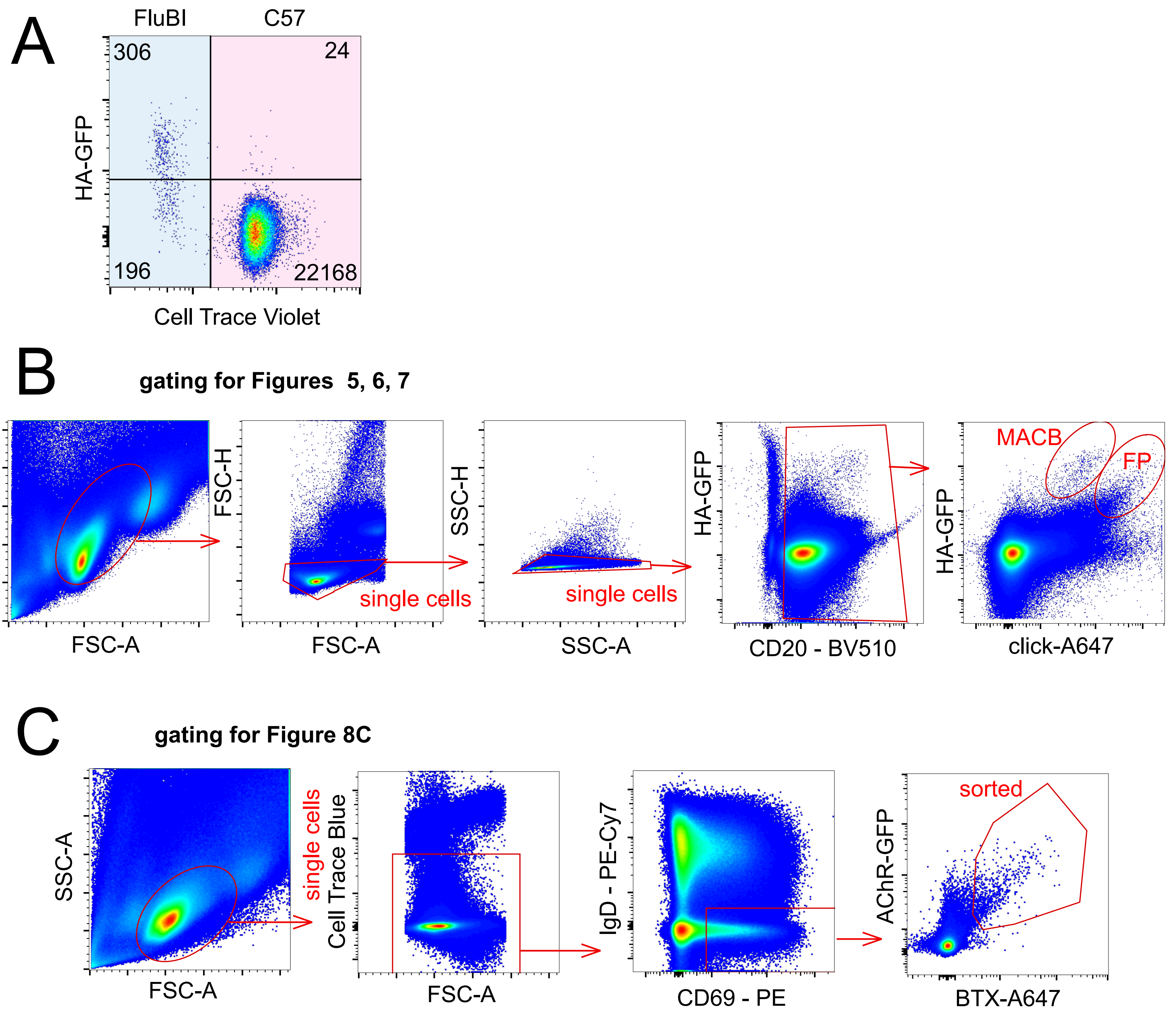

Supplementary Figure 3. Gating strategies. **(A)** Gating and calculation for extracellular quenching experiment shown in main Figure 1H. Main Figure 1H (as well as Supplementary Figure 4B and Supplementary Figure 5) reports the ratio of GFP-positive to GFP-negative cells within each B cell type. In the example shown here (non-quenched control) the ratio for FluBI B cells is 306/196 = 1.56; while the ratio for wild type C57 B cells = 24/22168 = 0.001. **(B)** Gating strategy for main Figures 5, 6 and 7. **(C)** Gating strategy for main Figure 8C.
