# Supplementary material for: Antigen Extraction and B Cell Activation Enable Identification of Rare Membrane Antigen Specific Human B Cells
Source: Front Immunol. 2019 Apr 16;10:829. doi: 10.3389/fimmu.2019.00829 (PMC6477023; doi:10.3389/fimmu.2019.00829)
Supplement: Supplementary file 5 [file Data_Sheet_4.PDF]

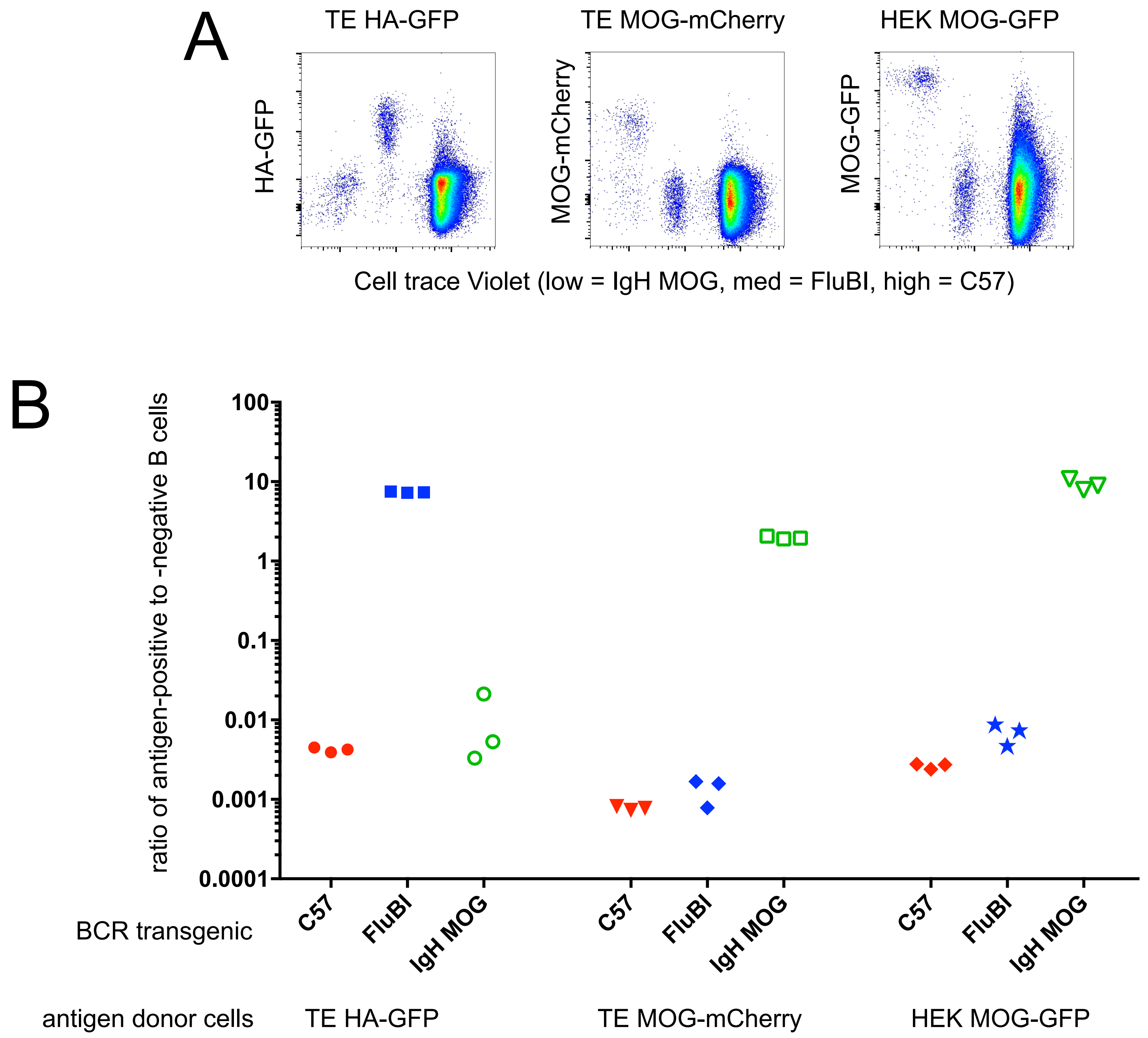

Supplementary Figure 4. Assessment of antigen acquisition by non-cognate BCR trangenic B cells. 400,000 wild type C57 B cells, mixed with 4,000 HA-specific FluBI B cells, and 4,000 myelin oligodendrocyte glycoprotein (MOG)-specific IgH MOG B cells were co-cultured with each of three different fluorescent antigen donor cell lines. The donor cell lines were TE cells expressing HA fused to GFP (TE HA-GFP, as used in main Figure 1D, etc); TE cells expressing MOG fused to mCherry (TE MOG-mCherry); and HEK cells expressing MOG fused to GFP (HEK MOG-GFP). The three different B cell types were labeled with different concentrations of Cell Trace Violet to allow us to distinguish them from one another after co-culture. For each B cell type, the numbers of cells that were positive for the fluorescent antigen after co-culture were compared. **(A)** Flow Cytometry dot plots showing fluorescent antigen levels for each of the three B cell types after co-culture. Gated on scatter and B220-positive. **(B)** Column scatter graph showing ratios of fluorescent antigen positive to negative B cells, grouped by the antigen donor cell line, and colored by the B cell type (red solid symbols = C57 wild type, blue solid symbols = FluBI, green outlines = IgH MOG
