# Supplementary material for: Antigen Extraction and B Cell Activation Enable Identification of Rare Membrane Antigen Specific Human B Cells
Source: Front Immunol. 2019 Apr 16;10:829. doi: 10.3389/fimmu.2019.00829 (PMC6477023; doi:10.3389/fimmu.2019.00829)
Supplement: Supplementary file 6 [file Data_Sheet_5.PDF]

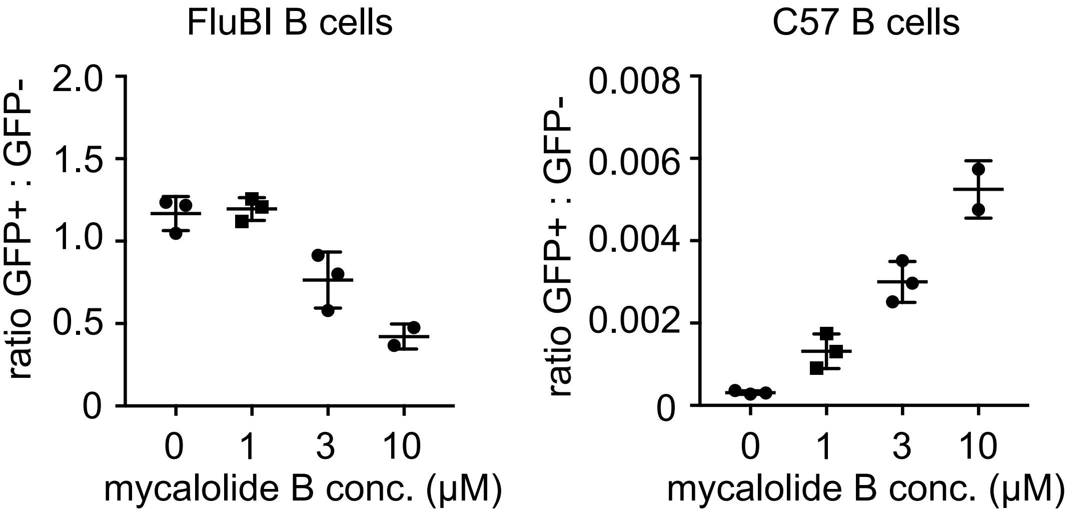

Supplementary Figure 5. Effect of actin depolymerization on cognate and non-cognate antigen acquisition. 4,000 FluBI B cells were mixed with 400,000 wild type C57 B cells, with the two cell types labeled with different concentrations of Cell Trace Violet. They were then added to adherent layers of TE cells expressing HA-GFP, that had been exposed to various concentrations of the actin depolymerizing agent mycalolide B (horizontal axis) and then washed with medium. After 1 hour of co-culture, the B cells were retrieved, and GFP fluorescence of the two cell types measured by flow cytometry. The vertical axis shows the ratio of GFP-positive B cells divided by GFP-negative cells. The left plot shows results for the HA-specific FluBI B cells, and the right plot shows results for the HA-irrelevant wild type C57 B cells.
