# Supplementary material for: Antigen Extraction and B Cell Activation Enable Identification of Rare Membrane Antigen Specific Human B Cells
Source: Front Immunol. 2019 Apr 16;10:829. doi: 10.3389/fimmu.2019.00829 (PMC6477023; doi:10.3389/fimmu.2019.00829)
Supplement: Supplementary file 7 [file Data_Sheet_6.PDF]

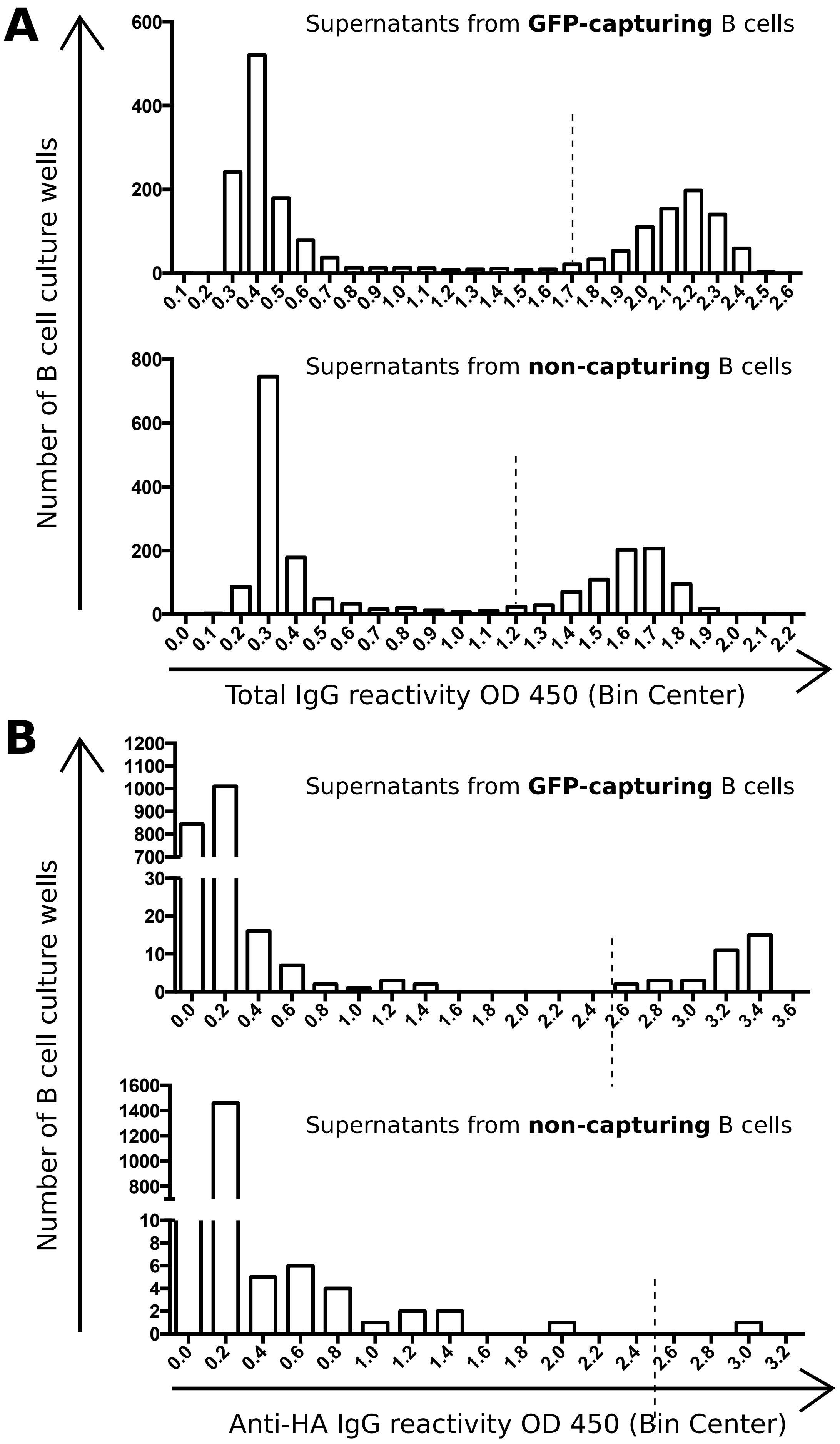

Supplementary Figure 6. Frequencies of antibody concentrations and hemagglutinin reactivities of single B cell culture supernatants. Frequencies of antibody concentrations and hemagglutinin reactivities of single B cell culture supeernatants. Related to Figure 3. **(A)** Histograms of the frequencies of supernatants of cultures derived from single HA-GFP-capturing (above) or non-capturing (below) B cells that contained IgG at concentrations yieleding the Optical Densities shown on the horizotal axis when measured by ELISA. **(B)** Exactly as A, but showing results produced using ELISA plates coated with recombinant influenza hemagglutinin, rather than anti-IgG capture antibody as used in (A).
