# Supplementary material for: Antigen Extraction and B Cell Activation Enable Identification of Rare Membrane Antigen Specific Human B Cells
Source: Front Immunol. 2019 Apr 16;10:829. doi: 10.3389/fimmu.2019.00829 (PMC6477023; doi:10.3389/fimmu.2019.00829)
Supplement: Supplementary file 8 [file Data_Sheet_7.PDF]

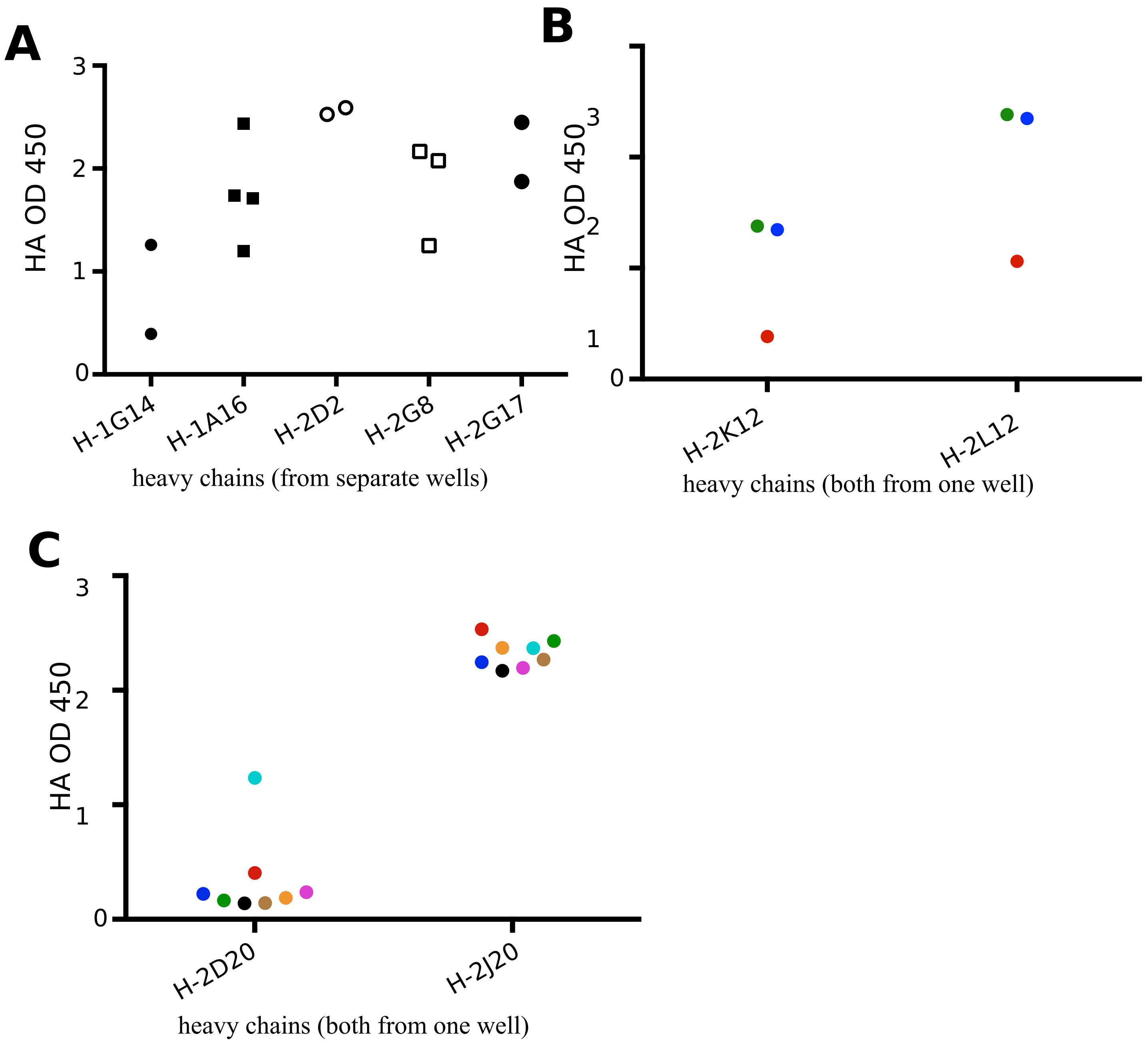

Supplementary Figure 7. Binding of recombinant monoclonal antibodies cloned from GFP-capturing B cell cultures (data points marked red in Figure 3). Different combinations of heavy and light chains were recombinantly expressed and tested by ELISA for their binding capacity to HA. The horizontal axes show the different heavy chains and the vertical axes show the HA OD 450. Binding OD for each combination is plotted on the vertical axis with a circle or square. (**A**) Five examples of heavy/light chain combinations originating from B cell culture wells containing only one single heavy chain (horizontal axis) and 2, 3 or 4 light chains. **(B)** Example of heavy/light chain combinations originating from one B cell culture well containing two heavy chains and three light chains (same light chains are indicated by color). **(C)** Example of heavy/light chain combinations originating from one B cell culture well containing two heavy chains and eight light chains (same light chains are indicated by color). .
