# Supplementary material for: Antigen Extraction and B Cell Activation Enable Identification of Rare Membrane Antigen Specific Human B Cells
Source: Front Immunol. 2019 Apr 16;10:829. doi: 10.3389/fimmu.2019.00829 (PMC6477023; doi:10.3389/fimmu.2019.00829)
Supplement: Supplementary file 9 [file Data_Sheet_8.PDF]

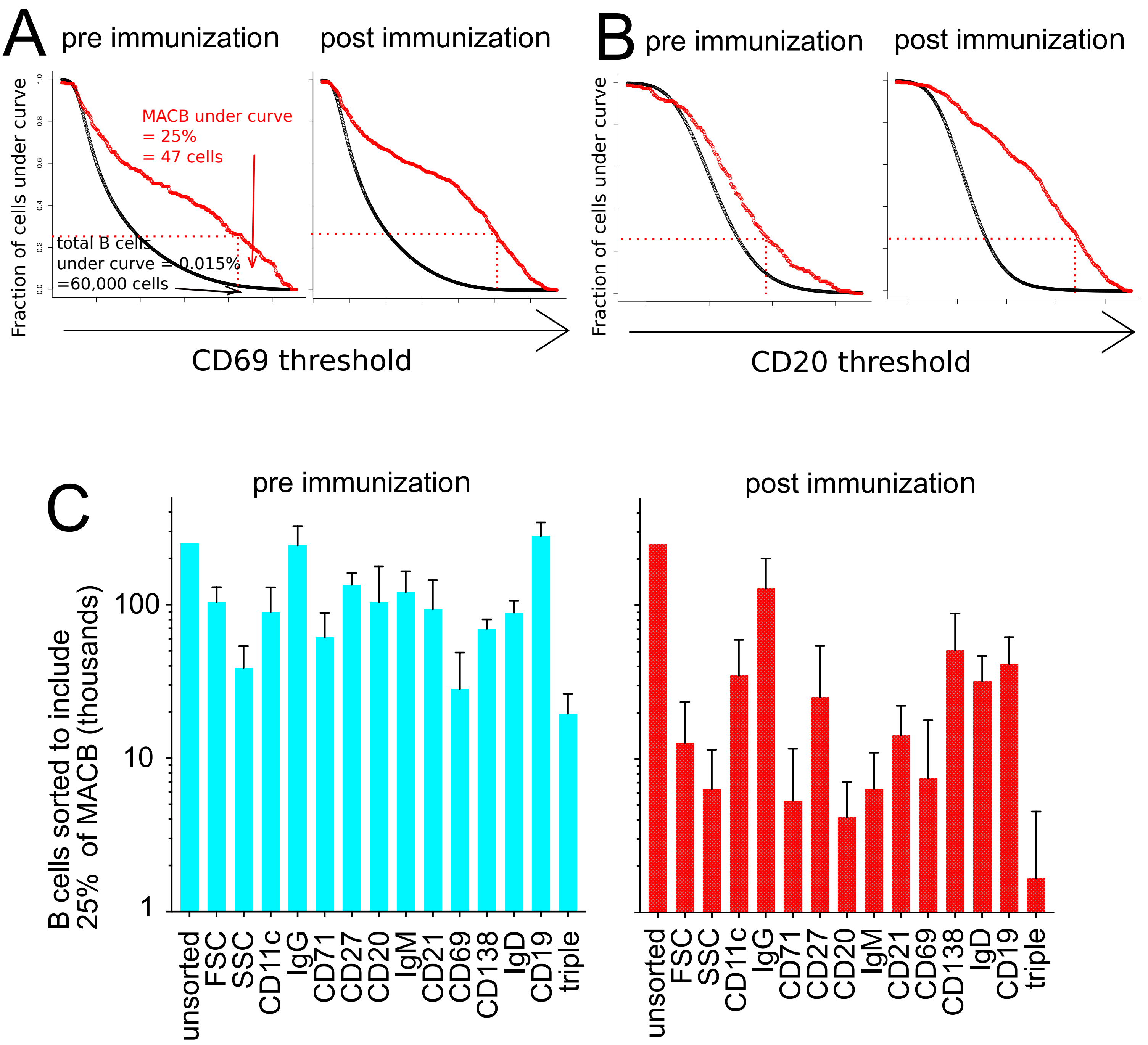

Supplementary Figure 8. Utility of single parameters in enriching antigen-specific B cells - (**A**) the example of CD69. Fractions of MACB (red curve) and total B cells (black curve) with CD69 expression above a given threshold are plotted against the range of possible thresholds. Horizontal broken red line is the 25% level of MACB inclusion used in the next figure. Comparison of plot on the left, taken from pre-immunization sample, and plot on the right from post-vaccination, show that sorting cells with high CD69 expression enables significant enrichment of antigen capturing cells at steady state, as well as after immunization. **(B)** Exactly analogous to (A), but showing CD20 expression, which differs between antigen-capturing B cells and the global B cell pool after immunization, but which is not very different before at steady state. **(C)** Estimates of the utility of each parameter in enriching for antigen-specific B cells. The vertical axis represents the number of total B cells that would have to be sorted to obtain 25% of the MACB cells as defined in the gate in Figure 4A, normalized to a total sample of 1 million B cells. For all but three parameters, the highest expressing cells were taken; for IgM, IgD, and CD21, the lowest cells were taken. The last column ("triple") is a combination of the three most promising markers, i.e., SSC, CD71, and CD69, obtained by multiplying the channel factors together and taking the cube root of the product.
